# Supplementary material for: A structural blueprint for interleukin-21 signal modulation
Source: Cell Rep. 2023 Jun 19;42(6):112657. doi: 10.1016/j.celrep.2023.112657 (PMC10320830; doi:10.1016/j.celrep.2023.112657)
Supplement: Document S1. Figures S1–S6 and Tables S1–S3 [file mmc1.pdf]

Cell Reports, Volume *42*

Supplemental information

A structural blueprint for interleukin-21 signal modulation

Gita C. Abhiraman, Theodora U.J. Bruun, Nathanael A. Caveney, Leon L. Su, Robert A. Saxton, Qian Yin, Shaogeng Tang, Mark M. Davis, Kevin M. Jude, and K. Christopher Garcia

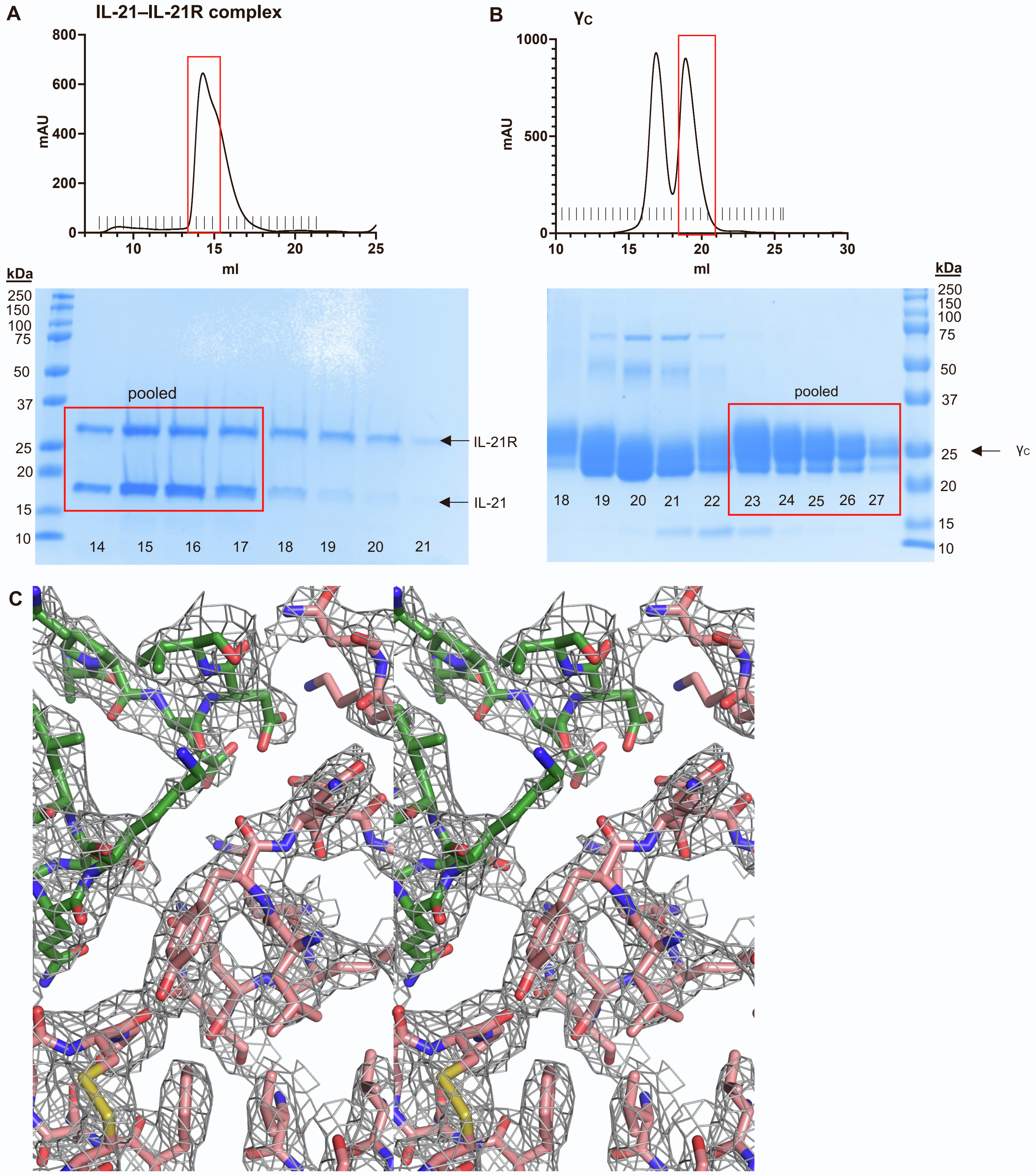

Figure S1. Structure of the IL-21 receptor complex, related to Figure 1

(A–B) Size exclusion chromatography UV traces for purified IL-21R and IL-21 (A) complex and γ_C (B) and accompanying Coomassie-stained SDS-PAGE gel. Fractions collected for crystallization trials are indicated in red. **(C)** Wall-eyed stereo view of site IIa interface. The final 2mFo-DFc map (shown in gray) is contoured at 1.0 σ. IL-21 shown in green, γc in pink.

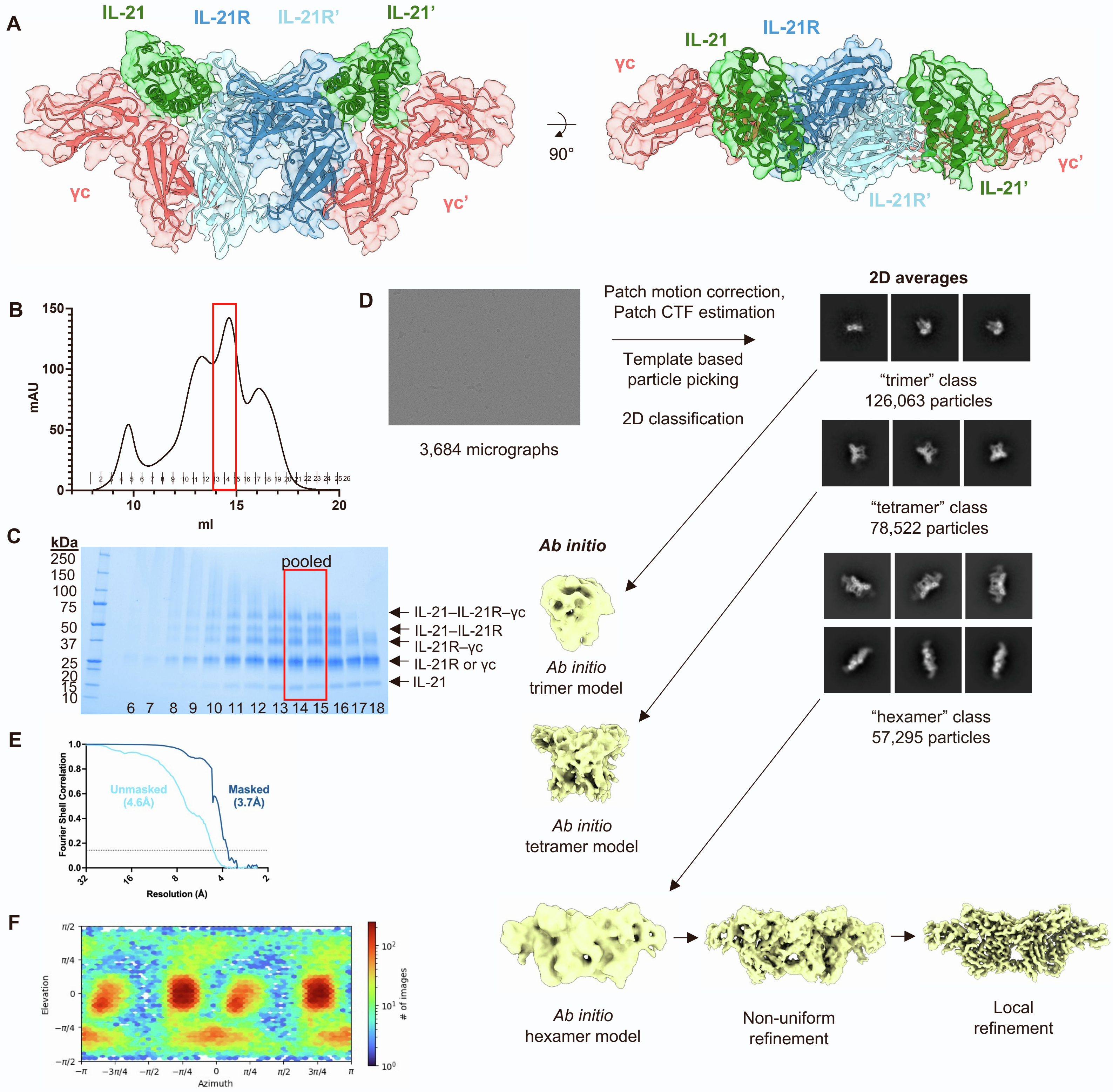

Figure S2. Cryo-electron microscopy of the IL-21 complex, related to Figure 1

(A) Final model of 2:2:2 IL-21:IL-21R:γ_C complex determined by cryoEM (shown in transparency, EMDB ID: EMD-28278) with crystallography model docked (shown as ribbon, PBD ID: 8ENT).

(B–C) Size exclusion chromatography UV trace for IL-21–IL-21R–γc complex after BS^3 cross-linking and accompanying Coomassie-stained SDS-PAGE gel. Fractions collected for cryo-EM studies are outlined in red.

(D) Cryo-EM data processing of the IL-21 complex, including representative micrograph from data acquisition. 2D class averages for hexamer, tetramer, and trimer classes and *ab initio* models generated for each class, followed by refinement scheme for the hexameric model.

(E) Fourier shell correlation (FSC) curve of final model.

(F) Orientational distribution of the complex reconstruction.

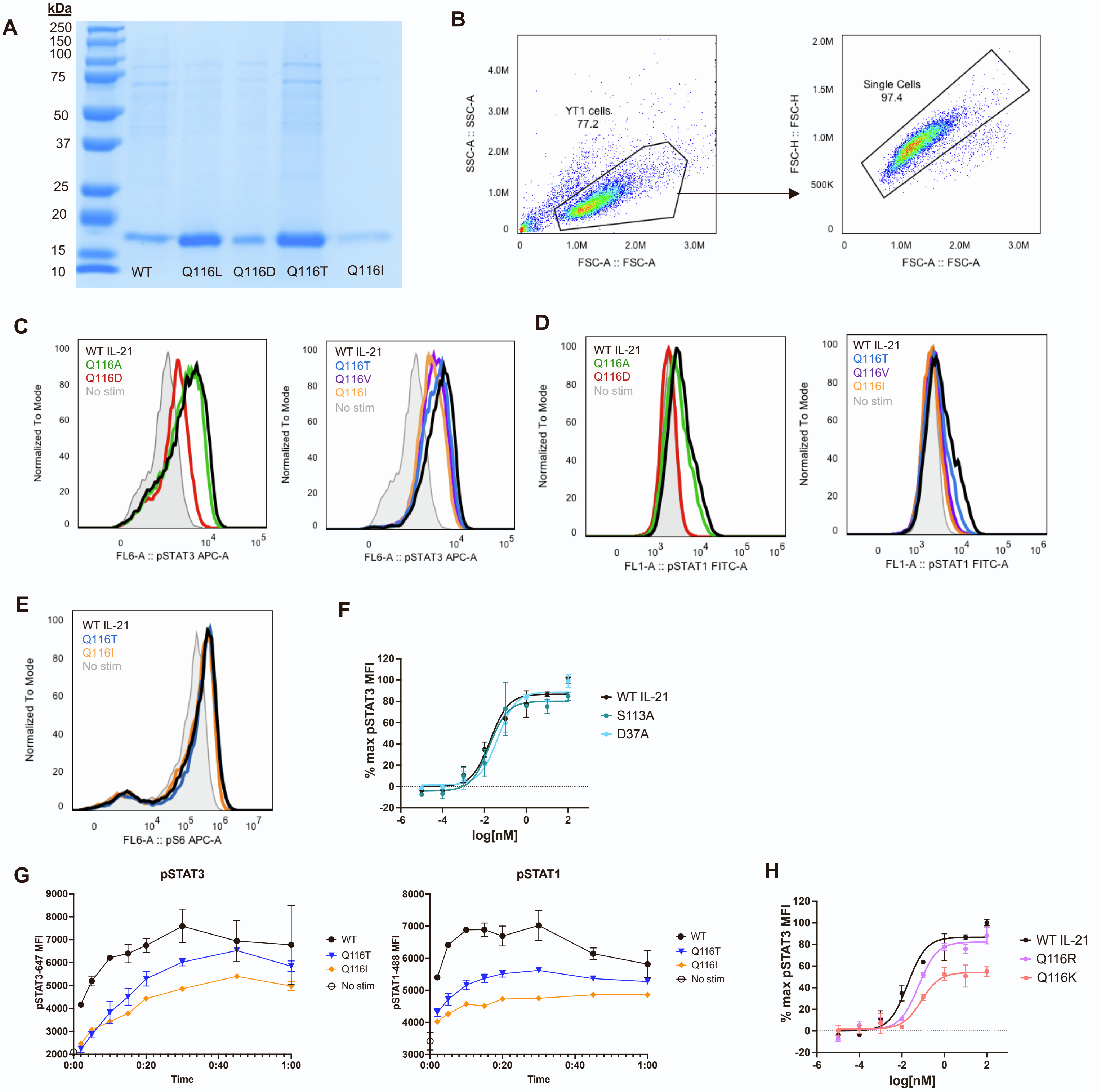

Figure S3. Design of human IL-21 partial agonists, related to Figure 3

(A) Coomassie-stained SDS-PAGE gel of His-tagged IL-21 variants purified by Ni-NTA.

(B) Representative gating on single YT-1 cells.

(C–D) Representative histograms of pSTAT3 (C) or pSTAT1 (D) intensity in YT-1 cell stimulated with wild-type IL-21 or variant.

(E) Representative histograms of pS6 intensity in YT-1 cells stimulated with wild-type IL-21 or variant.

(F) Dose-response curves for phopsho-STAT3 in YT-1 cells stimulated with wild-type IL-21 or the indicated variants for 20 minutes and analyzed by flow cytometry. Data are mean +/- SD for two replicates, shown as percent of maximal wild-type IL-21 MFI.

(G) Time-course signaling assays for intracellular phospho-STAT3 and phospho-STAT1 in YT-1 cells stimulated with 200nM IL-21 or variant for up to one hour. Data are mean +/- SD for two replicates.

(H) Dose-response curves for phopsho-STAT3 in YT-1 cells stimulated with wild-type IL-21 or the indicated variants for 20 minutes and analyzed by flow cytometry. Data are mean +/- SD for two replicates, shown as percent of maximal wild-type IL-21 MFI.

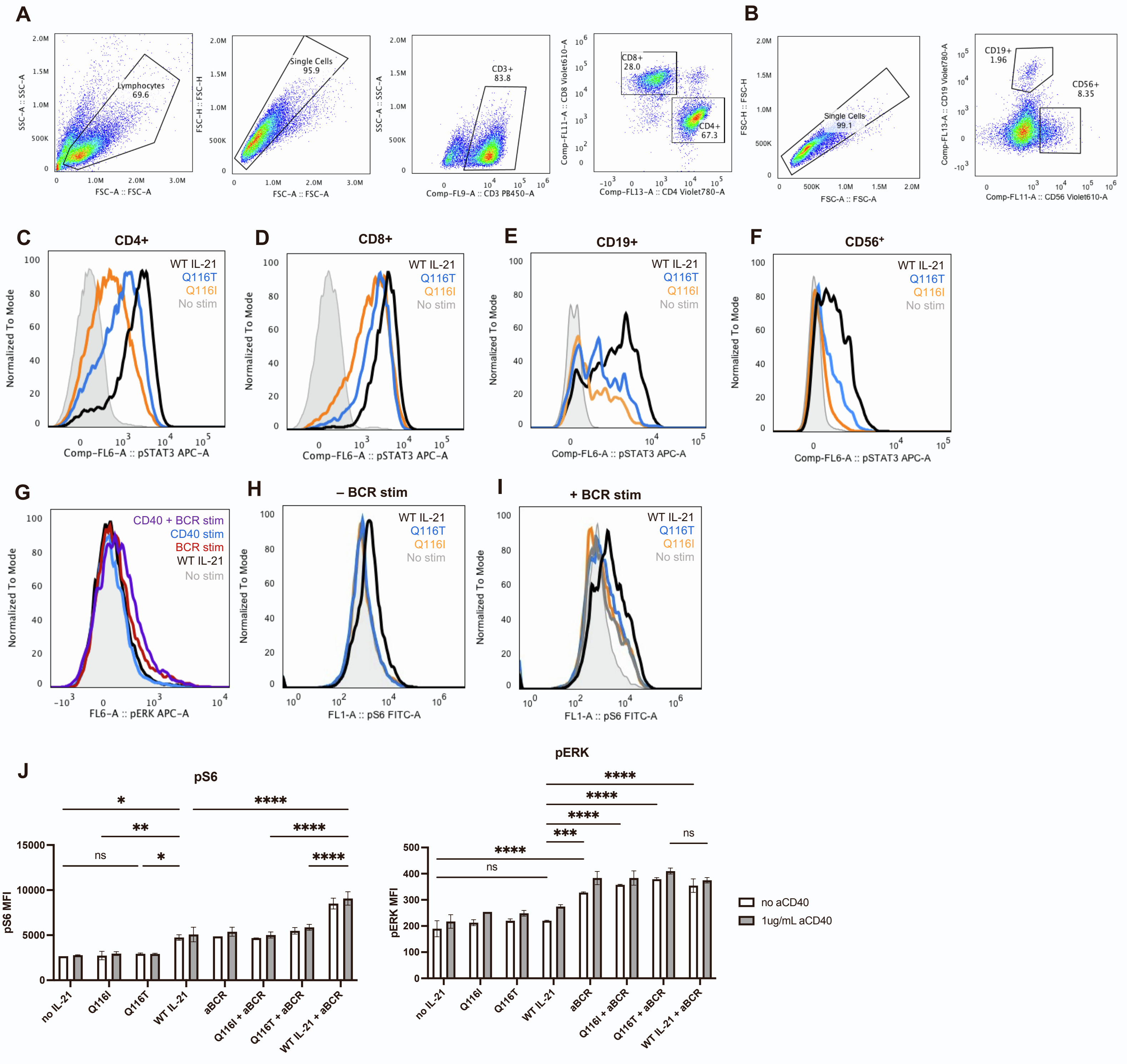

Figure S4. IL-21 signaling in primary human cells

(A–B) Representative gating of primary CD4⁺ and CD8⁺ T cells (A) or CD19⁺ and CD56⁺ cells from bulk human peripheral mononuclear cells (B).

(C–F) Representative histograms of pSTAT3 intensity in CD4⁺ (C), CD8⁺ (D), CD19⁺ (E) or CD56⁺ (F) cells stimulated with wild-type IL-21, Q116T, Q116I, or no cytokine.

(G) Representative histograms of pERK intensity of MACS isolated CD19⁺ cells stimulated with wild-type IL-21, BCR stimulation, or CD40 stimulation, corresponding to Figure 4B.

(H–I) Representative histograms of pS6 intensity of CD19⁺ cells stimulated with wild-type IL-21, Q116T, Q116I, or no cytokine with (H) or without (I) BCR stimulation, corresponding to Figure 4C.

(J) Bar graphs quantifying mean intensity of pS6 and pERK in B cells detected by flow cyometry, corresponding to heat maps in Figure 4B–C (N=2 biological replicates, * indicates $P \leq 0.05$, ** indicates $P \leq 0.01$, *** indicates $P \leq 0.001$, **** indicates $P \leq 0.0001$ by two-way ANOVA).

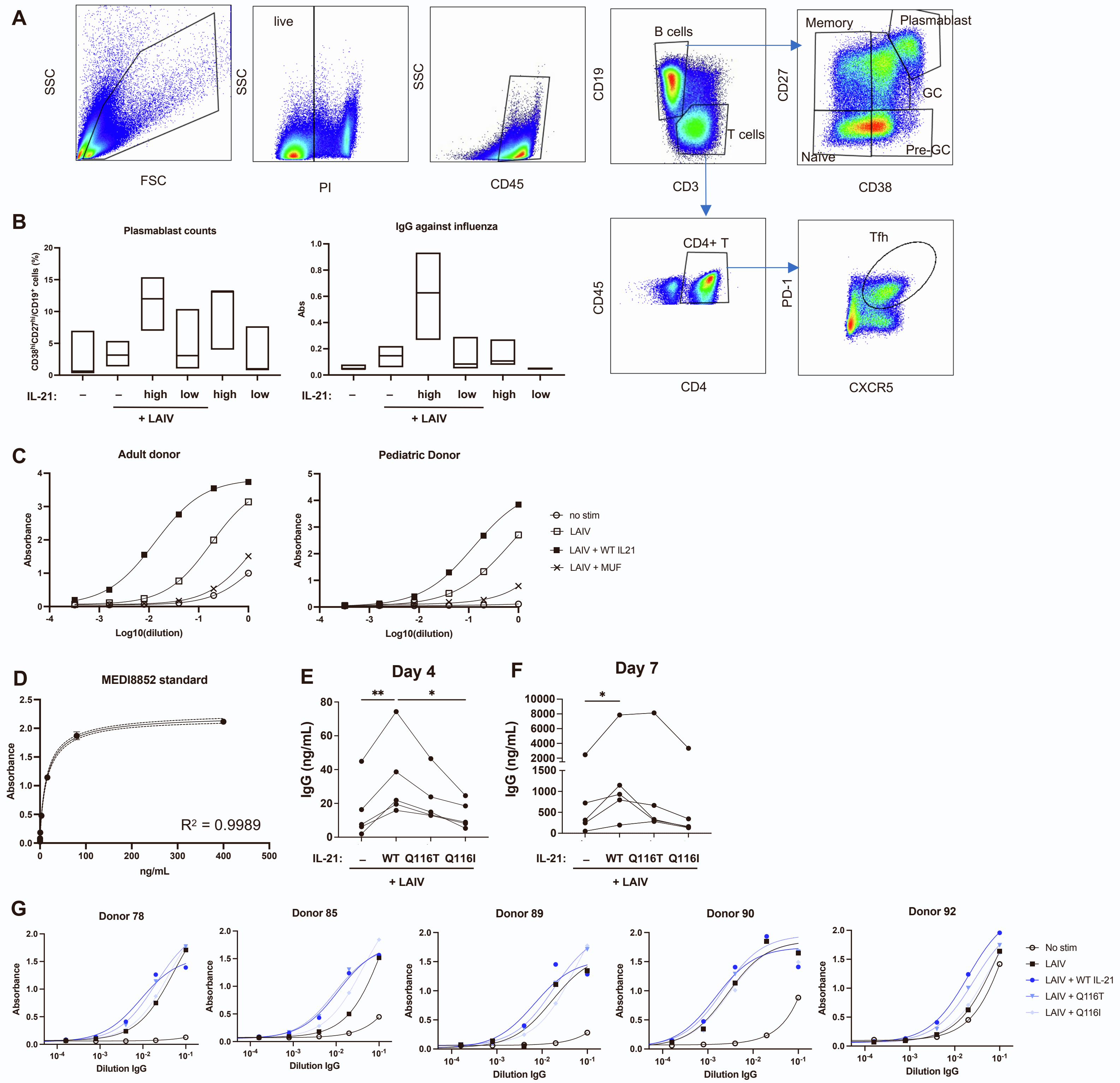

Figure S5. Characterization of IL-21 response in human tonsil organoids, related to Figure 4

(A) Representative gating of naïve B cells, memory B cells, germinal center B cells (GC), pre-germinal center B cells (pre-GC), plasmablasts, and T follicular helper (Tfh) cells in human tonsil organoids.

(B) Plasmablast frequency (% of total B cells) and flu-specific IgG detected by ELISA from tonsil organoids cultured for 7 days with or without live attenuated influenza virus (LAIV) and 50ng/ml (high dose) or 10ng/mL (low dose) IL-21 (N=3 human donors).

(C) Representative titrations of flu-specific IgG detected by ELISA in human tonsil organoids from an adult and pediatric donor.

(D) Representative standard curves derived from broadly neutralizing antibody MEDI8852 binding to whole flu used for quantification of flu-specific antibodies in human tonsil experiments.

(E–F) Flu-specific IgG quantified by ELISA from tonsil organoids vaccinated with LAIV and indicated IL-21 variant on day 4 (F) and day 7 (G). IgG was quantified using broadly neutralizing flu antibody. Raw IgG values plotted as ng/mL. * indicates P ≤ 0.05, ** indicates P ≤ 0.01 by ANOVA. (N=5 human donors.)

(G) Titrations of flu-specific IgG detected by ELISA in human tonsil organoids derived from 5 donors, stimulated with LAIV, wild-type IL-21, Q116T, or Q116I.

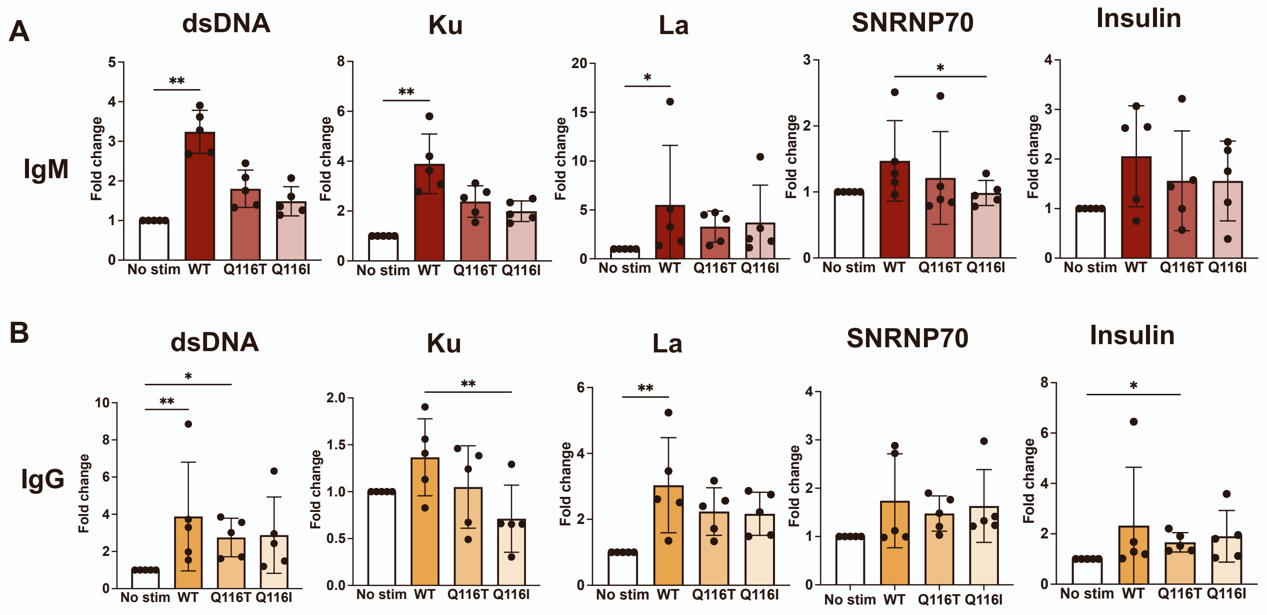

Figure S6. Autoantibody production is modulated by IL-21 variants

(A–B) IgM (A) and IgG (B) against self-antigens dsDNA, Ku and La, quantified by ELISA on day 7 in tonsils cultured with 100nM IL-21 or variant. Data are mean +/- SD for five human donors.
* indicates P ≤ 0.05, ** indicates P ≤ 0.01 by one-way ANOVA.

Table S1. Data collection and refinement statistics, related to Figure 1

Protein	IL-21–IL-21R– γc complex
PDB ID	8ENT
<u>Data collection</u>	
Wavelength	0.9795
Resolution range (Å)	48.17 - 2.831 (2.932 - 2.831)
Space group	P1
a, b, c (Å)	66.167 66.464 162.684
α, β, ɣ	83.338 83.708 73.098
Total reflections	211574 (20618)
Unique reflections	57754 (5757)
Multiplicity	3.7 (3.6)
Completeness (%)	91.66 (88.39)
Mean I/sigma	4.22 (0.66)
R_{sym}	0.2143 (1.636)
R_{meas}	0.2506 (1.914)
R_{pim}	0.1292 (0.9883)
$CC_{1/2}$	0.985 (0.401)
CC*	0.996 (0.757)
<u>Refinement</u>	
Reflections used in refinement	57397 (5529)
Reflections used for R-free	1963 (194)
R_{work}	0.2784 (0.4586)
R_{free}	0.3120 (0.4967)
Number of non-hydrogen atoms	15852
macromolecules	15520
ligands	277
solvent	55
Protein residues	1888
RMS(bonds) (Å)	0.002
RMS(angles) (°)	0.6
Ramachandran favored (%)	94.72
Ramachandran allowed (%)	5.28
Ramachandran outliers (%)	0
Rotamer outliers (%)	3.76
Clashscore	13.37
Average B-factors:	74.21
macromolecules	74.11
ligands	84.98
solvent	48.56

Table S2. Key contacts in IL-21 receptor complex

Site IIa<br>IL-21–IL-21R interface			
**Hydrogen Bonds**	**γc**	**Dist. [Å]**	**IL-21**
1	F:THR 105 [Oγ]	2.75	D:ASP 37 [Oδ]
2	F:GLN 127 [Nε]	3.12	D:SER 113 [Oγ]
3	F:GLN 127 [Oε]	3.52	D:GLN 116 [Nε]

Site IIb<br>IL-21R–γc interface			
**Hydrogen Bonds**	**γc**	**Dist. [Å]**	**IL-21R**
1	F:ARG 183 [Nη]	3.17	E:GLU 136 [Oε]
2	F:SER 146 [Oγ]	2.81	E:GLU 170 [Oε]
3	F:GLN 147 [Nε]	2.77	E:LEU 156 [O]
4	F:SER 187 [Oγ]	3.13	E:SER 158 [Oγ]
5	F:SER 187 [Oγ]	2.78	E:LEU 156 [O]
6	F:GLN 147 [Oε]	3.12	E:LEU 156 [N]
7	F:SER 187 [Oγ]	3.72	E:SER 158 [N]
8	F:SER 190 [Oγ]	2.83	E:LEU 167 [N]
9	F:TYR 182 [Oη]	2.70	E:LYS 134 [Nζ]

Site III<br>IL-21R–IL-21R interface			
**Hydrogen Bonds**	**IL-21R**	**Dist. [Å]**	**IL-21R**
1	H:HIS 24 [Nδ]	3.20	E:ASP 125 [Oδ]
2	H:HIS 53 [Nε]	2.43	E:ASP 122 [Oδ]
3	H:TRP 148 [N]	3.17	E:ARG 144 [O]
4	H:TRP 148 [N]	2.91	E:ASP 146 [O]
5	H:TRP 148 [Nε]	2.55	E:GLU 178 [Oε]
6	H:ALA 149 [N]	2.90	E:ARG 144 [O]
7	H:THR 61 [Oγ]	2.92	E:HIS 63 [Nε]
8	H:ASP 122 [Oδ]	2.70	E:HIS 53 [Nε]
9	H:ASP 125 [Oδ]	3.00	E:HIS 24 [Nδ]
10	H:ARG 144 [O]	3.10	E:TRP 148 [N]
11	H:ARG 144 [O]	2.67	E:ALA 149 [N]
12	H:ASP 146 [O]	2.73	E:TRP 148 [N]
13	H:GLU 178 [Oε]	2.77	E:TRP 148 [Nε]

Site III<br>IL-21R–IL-21R interface			
**Salt Bridges**	**IL-21R**	**Dist. [Å]**	**IL-21R**
1	H:HIS 24 [Nδ]	3.20	E:ASP 125 [Oδ]
2	H:HIS 53 [Nε]	2.43	E:ASP 122 [Oδ]
3	H:ASP 122 [Oδ]	2.70	E:HIS 53 [Nε]
4	H:ASP 122 [Oδ]	2.95	E:HIS 53 [Nε]
5	H:ASP 125 [Oδ]	3.00	E:HIS 24 [Nδ]

Table S3. Cryo-EM data collection, refinement, and validation statistics

Protein	IL-21–IL-21R– γc complex
**EMD**	EMD-28278
**Data collection and processing**	
Magnification	45,000
Voltage (keV)	200
Electron exposure ($e^-/Å^2$)	50
Defocus range (μm)	-1.0 to -2.5
Pixel size (Å)	1.15
Symmetry imposed	C2
Initial particle images	1,444,277
Final particle images	57,295
Map resolution FSC threshold (Å)	0.143
Map resolution (Å)	3.7
**Model Fitting**	
Model used (PDB)	8ENT
Model resolution FSC threshold (Å)	0.5
Model resolution (Å)	4.2
