# Supplementary material for: A structural blueprint for interleukin-21 signal modulation
Source: Cell Rep. 2023 Jun 19;42(6):112657. doi: 10.1016/j.celrep.2023.112657 (PMC10320830; doi:10.1016/j.celrep.2023.112657)
Supplement: Document S2. Article plus supplemental information [file mmc2.pdf]

Cell Reports

Article

A structural blueprint for interleukin-21 signal modulation

Graphical abstract

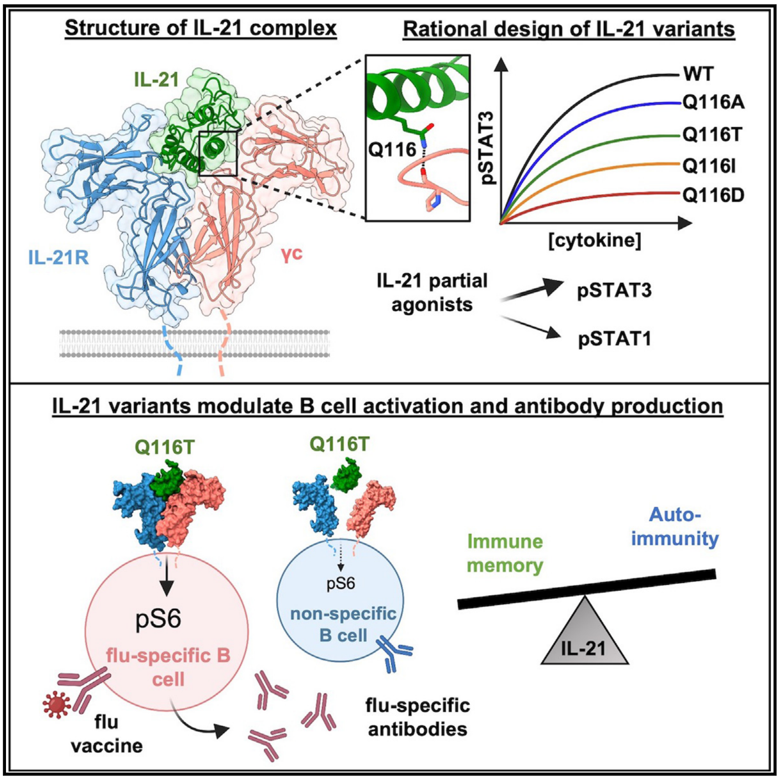

Highlights

- Crystal structure and cryo-EM reconstruction of the IL-21 receptor complex
- Structural comparison of common-gamma family cytokines
- Engineered IL-21 variants modulate pS6, pSTAT3, and pSTAT1 signaling
- IL-21 variants enhance flu-specific antibody production in tonsil organoids

Authors

Gita C. Abhiraman, Theodora U.J. Bruun, Nathanael A. Caveney, ..., Mark M. Davis, Kevin M. Jude, K. Christopher Garcia

Correspondence

kcgarcia@stanford.edu

In brief

Abhiraman et al. solve structures of the IL-21 receptor complex by X-ray crystallography and cryo-electron microscopy. Structure-based engineering of IL-21 leads to variants that modulate pS6, pSTAT3, and pSTAT1 signaling. Engineered IL-21 variants enhance flu-specific antibody production in human tonsil organoids, with reduced off-target antibody production.

Abhiraman et al., 2023, Cell Reports *42*, 112657
June 27, 2023 © 2023 The Author(s).
https://doi.org/10.1016/j.celrep.2023.112657

CellPress

Cell Reports

CellPress
OPEN ACCESS

Article

A structural blueprint for interleukin-21 signal modulation

Gita C. Abhiraman,[1,2] Theodora U.J. Bruun,[3,4] Nathanael A. Caveney,[1] Leon L. Su,[1] Robert A. Saxton,[1] Qian Yin,[5,6] Shaogeng Tang,[3,4] Mark M. Davis,[5,6] Kevin M. Jude,[1,6] and K. Christopher Garcia[1,6,7,*]
[1]Department of Molecular and Cellular Physiology, Stanford University School of Medicine, 279 Campus Drive, Stanford, CA 94305, USA
[2]Program in Immunology, Stanford University School of Medicine, Stanford, CA 94305, USA
[3]Department of Biochemistry, Stanford University School of Medicine, Stanford, CA 94305, USA
[4]Sarafan ChEM-H, Stanford University, Stanford, CA 94305, USA
[5]Institute for Immunity, Transplantation and Infection, Stanford University School of Medicine, Stanford, CA 94305, USA
[6]Howard Hughes Medical Institute, Stanford University, Stanford, CA 94305, USA
[7]Lead contact
*Correspondence: kcgarcia@stanford.edu
https://doi.org/10.1016/j.celrep.2023.112657

SUMMARY

Interleukin-21 (IL-21) plays a critical role in generating immunological memory by promoting the germinal center reaction, yet clinical use of IL-21 remains challenging because of its pleiotropy and association with autoimmune disease. To better understand the structural basis of IL-21 signaling, we determine the structure of the IL-21–IL-21R–γc ternary signaling complex by X-ray crystallography and a structure of a dimer of trimeric complexes using cryo-electron microscopy. Guided by the structure, we design analogs of IL-21 by introducing substitutions to the IL-21–γc interface. These IL-21 analogs act as partial agonists that modulate downstream activation of pS6, pSTAT3, and pSTAT1. These analogs exhibit differential activity on T and B cell subsets and modulate antibody production in human tonsil organoids. These results clarify the structural basis of IL-21 signaling and offer a potential strategy for tunable manipulation of humoral immunity.

INTRODUCTION

IL-21 is a highly pleiotropic common-gamma-chain cytokine that exhibits diverse actions on a broad range of immune cell types including T, B, and natural killer (NK) cells.[1] IL-21 promotes cytotoxicity of CD8$^+$ T cells and NK cells and has been evaluated as a cancer therapy in phase I and II clinical trials.[2] IL-21 is also critical for the formation of immunological memory, due to its actions on T follicular helper cells and B cells to promote the germinal center reaction.[3–5] IL-21 secretion by T follicular helper cells is essential for B cell activation, plasma cell formation, and class switch recombination, which are essential for lasting antibody protection after infection or vaccination.[6]

A major challenge to the use of IL-21 clinically is its association with the induction of autoimmune disease.[1] IL-21 has been investigated preclinically for its ability to stimulate antiviral immunity and antitumor responses.[2,7,8] However, IL-21 is also implicated in multiple autoimmune diseases including systemic lupus erythematosus, rheumatoid arthritis, multiple sclerosis, and inflammatory bowel disease.[9–11] IL-21 was found to drive the expansion of autoreactive plasma cells in human lupus patients.[12] Conversely, blockade of IL-21 signaling reduced disease progression in lupus-prone mice.[13] In gut inflammatory disorders, IL-21 produced by T helper 17 (Th17) cells is thought to drive IL-17 production and help maintain a Th17 lineage.[14,15] These potential adverse effects of IL-21 signaling raise the question of whether IL-21 can be leveraged for its potentially beneficial therapeutic uses without non-specific immune activation.

Our ability to rationally modulate IL-21 biology was limited by lack of complete structural information about how the cytokine binds to its receptors and assembles in its complete signaling complex. IL-21 is a member of the common-gamma (γc) family of cytokines, whose other members include IL-2, IL-4, IL-7, IL-9, and IL-15. IL-21 was predicted to signal through a heterodimeric receptor: it binds with high affinity to IL-21 receptor (IL-21R) and with low affinity to the shared γc receptor.[1] The dimerization of IL-21R and γc leads to the transphosphorylation of JAK1 and JAK3, which in turn phosphorylate and activate transcription factor STAT3 and, to a lesser extent, STAT1 and STAT5. In addition to the JAK/STAT pathway, IL-21 also activates the phosphoinositide 3-kinase (PI3K) and mitogen-activated protein kinase (MAPK) pathways.[16] Although a structure of a partial complex containing IL-21 bound to IL-21R has been reported,[17] the structure of the IL-21 signaling complex with shared receptor γc remains unsolved, limiting insight into mechanisms of IL-21 pleiotropy and avenues for cytokine engineering.

Here, we determined a 2.8 Å crystal structure and 3.7 Å cryogenic electron microscopy (cryo-EM) structure of the IL-21–IL-21R–γc complex, revealing the assembly of the IL-21 receptor complex and the basis of γc cytokine family cross-reactivity. Guided by the structure, we designed a series of human IL-21

© 2023 The Author(s).
This is an open access article under the CC BY license (http://creativecommons.org/licenses/by/4.0/).

analogs that modulated the induction of downstream phospho-S6 and phospho-STAT signaling. Given the important role for IL-21 in the germinal center reaction, we tested the effects of IL-21 and our engineered ligands on antibody production in human tonsil organoids. IL-21 analogs induced a range of B cell activation and antibody production. Taken together, these results reveal the structural basis for IL-21 signaling and avenues for IL-21 engineering in humoral immune activation.

RESULTS

Structure of the ternary IL-21 receptor complex

We pursued X-ray crystallographic studies to determine the IL-21 signaling complex with IL-21R and γc receptor. We first co-purified a glycan-reduced variant of IL-21 (N68Q) bound to a glycan-reduced variant of IL-21R (N78Q/N85Q/N106D/N116Q) by size-exclusion chromatography (Figure S1A). Residue numbering of IL-21 corresponds to the mature peptide as described, beginning with Gln1 after the 30-residue signal peptide.[18] The addition of N-terminally truncated γc to the IL-21–IL-21R complex enabled crystallization of the ternary complex (Figure S1B). Solution of the structure by molecular replacement at 2.8 Å resolution revealed an asymmetric unit containing three ternary IL-21–IL-21R–γc complexes and a fourth complex of IL-21–IL-21R, in which binding of the γc subunit is blocked by a symmetry-related molecule in the crystal. In this fashion, we resolved the complete ternary IL-21 signaling complex comprising IL-21, a four-helix bundle cytokine, bound to receptors IL-21R and γc (Figures 1A and 1B).

The overall architecture of the ternary complex bears similarity to other γc family cytokines including IL-2, IL-4, and IL-15, in which the cytokine bridges the receptor heterodimers in a Y-shaped fork.[19] At site I, similar to the previously solved binary complex structure,[17] IL-21 engages the private receptor IL-21R D1 domain through helix A and helix C, burying a combined surface area of 1,003 Å^2. Because we expressed IL-21R in insect cells, we did not observe C-mannosylation at the WSXWS motif as previously reported.[17] Our structure additionally reveals the IL-21–γc interface at site IIa, in which IL-21 engages γc (D1) through helix D and the AB loop, burying 605 Å^2 (Figure 1C). In IL-21, residue D37 on IL-21 (denoted D37$^{IL\text{-}21}$) in the AB loop forms a hydrogen bond with T105$^{\gamma c}$. Residues S113$^{IL\text{-}21}$ and Q116$^{IL\text{-}21}$ of helix D form hydrogen bonds with Q127$^{\gamma c}$ and the backbone carbonyl at P207$^{\gamma c}$ (Figure 1C and Table S2). Extensive receptor-receptor contacts comprise the site IIb "stem" interface between IL-21R and γc (Figure 1D). The interface is stabilized by numerous hydrogen bonds between the D2 domains of each receptor (Figure 1D and Table S2). The buried surface area of this stem interface is 750 Å^2, similar to that seen in the IL-2 and IL-4 receptor complexes.[20,21]

Structure of a hexameric IL-21 receptor complex

In the asymmetric unit of the crystal structure, an IL-21R–IL-21R homodimer bridges two copies of the IL-21 ternary complex, forming a hexameric (dimer of trimers) complex not observed for other γc cytokines (Figure S2A). We found that the same IL-21R–IL-21R site interaction was also present in the crystal lattice of a previously determined structure of the binary IL-21–IL-21R complex.[17] To further clarify the stoichiometry of the receptor complex using a non-crystallographic method, we pursued cryo-EM studies of the IL-21 receptor complex (Figures S2B–S2E). We found that the dimer of IL-21–21R–γc trimers was persistent in the cryo-EM micrographs. Using *ab initio* modeling and subsequent refinement, we determined a 3.7 Å resolution reconstruction of a hexameric IL-21 complex containing the same IL-21R–IL-21R dimeric interface (Figures 1E and 1F). Our crystal structure model docked into the cryo-EM map, showing that the 2:2:2 complexes observed in crystallography and in cryo-EM are consistent (Figure S2). The IL-21R–IL-21R interface is extensive, burying 1,334 Å^2 and mediated by over 18 key contacts (Table S2), including salt bridges between H24 and H53 on one copy of IL-21R with D125 and D122 on the second copy of IL-21R (Figure 1G), and a stacking interaction between W148 and R142 of each IL-21R (Figure 1G).

Structural basis of cytokine receptor sharing across γc family cytokines

A comparison of the ternary IL-21 complex structure with other γc family cytokines clarifies the structural basis for cross-reactivity among γc family cytokines IL-2, IL-4, IL-15, and IL-21. Relative to site I, the γc cytokine site IIa interface has a smaller surface area and poorer shape complementarity, in keeping with the role of γc as a degenerate receptor for multiple ligands. We analyzed a structural superposition of the IL-21 complex with three previously determined structures of γc cytokine complexes.[20–22] This structural comparison shows that the site IIa interaction for IL-2, IL-4, IL-15, and IL-21 is principally mediated through helix D (Figure 2A). IL-21 diverges from other γc cytokines at helix D in site IIa, where IL-21 is angled away from γc at the C terminus compared with IL-2, IL-4, and IL-15 (Figure 2B).

Inspection of key site IIa residues on IL-2 and IL-21 highlights a difference in the pitch of helix D and alteration in the context of contact with a shared "hotspot" glutamine residue: Q116$^{IL\text{-}21}$ and Q126$^{IL\text{-}2}$ (Figures 2C–2E). A sequence alignment of all γc cytokines reveals high conservation at this residue, with four out of six family members sharing a glutamine at this position on helix D (Figure 2D). In the case of IL-4, this glutamine is substituted with an arginine, which retains hydrogen bonding with the P207$^{\gamma c}$ backbone (Figure 2E). In addition, there is conservation of a glutamate on helix D (E106$^{IL\text{-}21}$) and an aspartate on helix B (D37$^{IL\text{-}21}$) across four of six family members (Figure 2D). In the case of the helix B interaction with γc, there is conserved hydrogen bonding between T105$^{\gamma c}$ with D37$^{IL\text{-}21}$ or D30$^{IL\text{-}15}$, yet there are no notable interactions between helix B and γc in the IL-2 and IL-4 structures (Figure 2F). Although the site IIa interface is defined by relatively few interactions compared with site I and site IIb, there is a high degree of conservation of the key helix D residues, underscoring the evolutionary pressure for receptor sharing in the γc cytokine family.

Structure-based design of IL-21 partial agonists

A major challenge in deploying IL-21 therapeutically is harnessing its immunostimulatory effects while minimizing off-target autoimmunity. Previous studies on pleiotropic cytokines such as IL-2, IL-10, and IL-12 have employed cytokine engineering at the low-affinity site II interface as a means of titrating the

CellPress
OPEN ACCESS

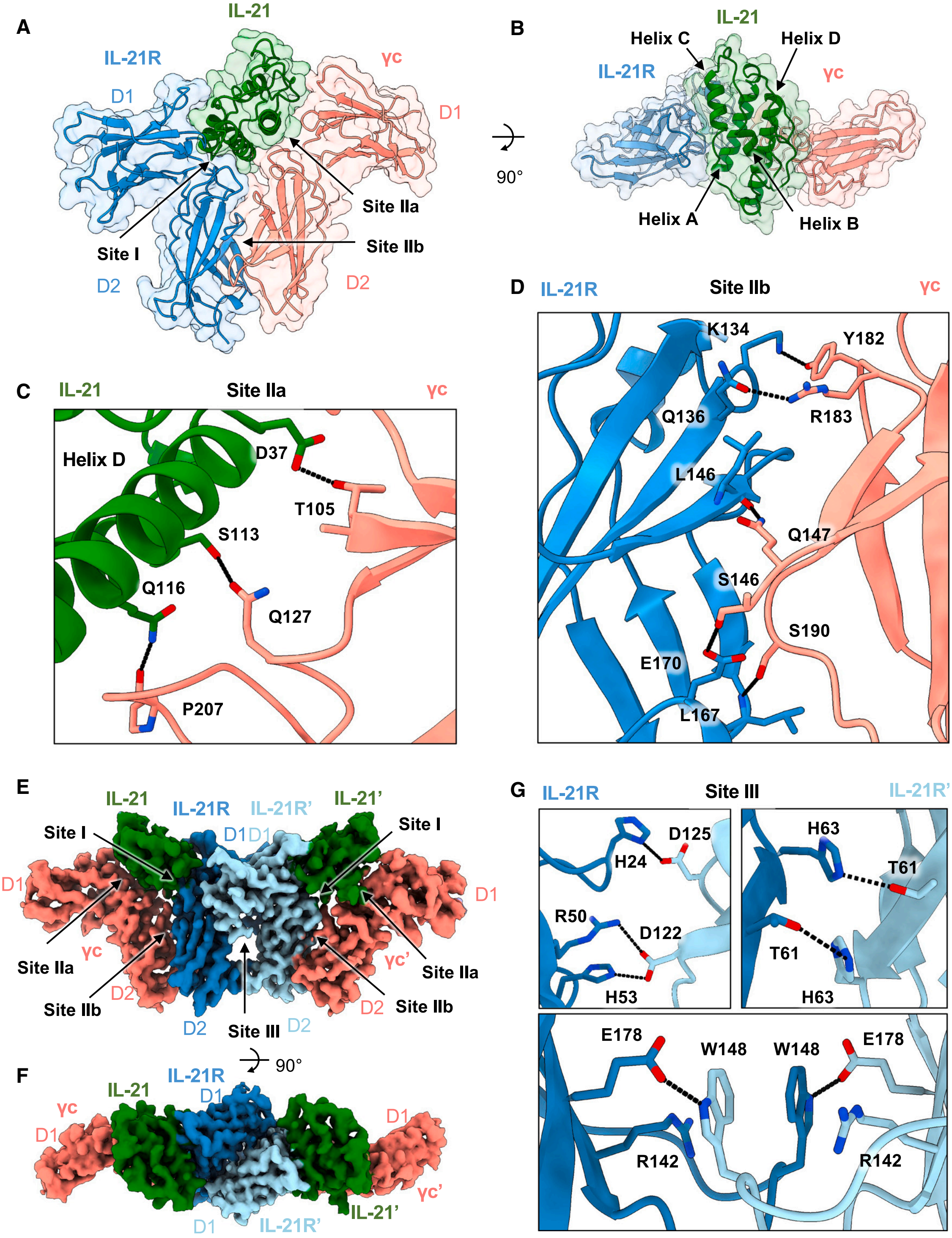

Figure 1. Structure of the IL-21 receptor complex

(A and B) Two views of the 2.8 Å resolution structure of the ternary IL-21 receptor complex, showing IL-21 in green, IL-21R in blue, and γc in pink (PDB: 8ENT).

(C) Close-up view of the IL-21–γc binding interface at site IIa. Hydrogen bonds are shown as black dashed lines.

(D) Close-up view of the IL-21R–γc binding interface at site IIb. Hydrogen bonds are shown as black dashed lines.

(E and F) Two views of the 3.7 Å resolution reconstruction of the 2:2:2 IL-21–IL-21R–γc complex determined by cryo-EM and retaining the IL-21R–IL-21R interface observed in the crystal structure (EMDB: EMD-28278).

(G) Close-up views of key contacts in the IL-21R–IL-21R site III interface.

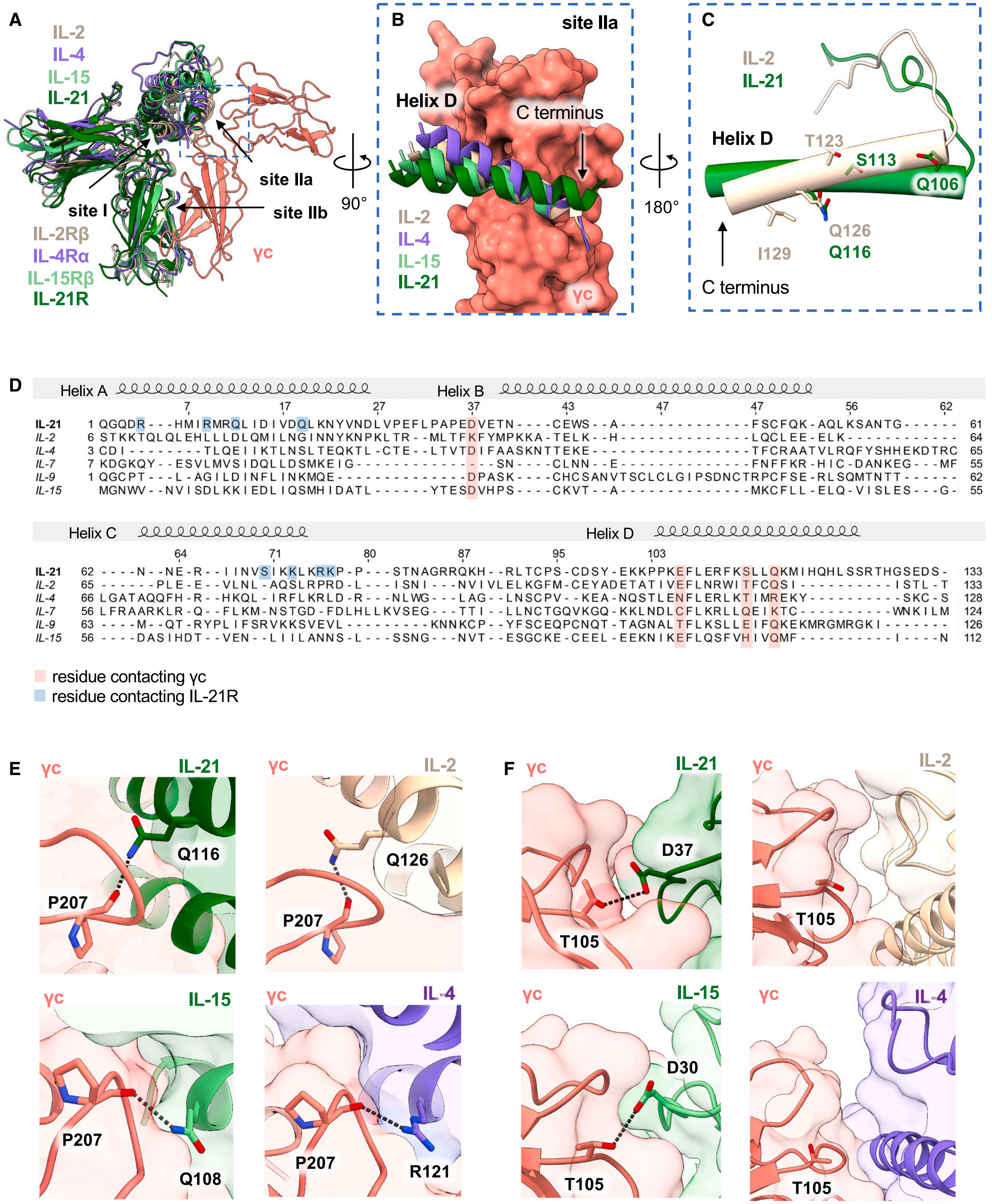

Figure 2. Structural basis for common-gamma family receptor sharing

(A) Structural alignment of the IL-21 ternary complex with IL-2 shown in beige (PDB: 2B5I), IL-4 in purple (3BPL), and IL-15 in light green (4GS7).

(B) Surface representation of γc (pink, right), comparing the site IIa interaction with IL-2 (beige), IL-4 (purple), IL-15 (light green), and IL-21 (dark green).

(legend continued on next page)

recruitment efficiency of γc in order to tune downstream signaling pathways.[23–26] We wished to determine whether tuning IL-21 affinity through amino acid substitutions at the site IIa interface with γc receptor could serve to modulate downstream activation of the pSTAT, PI3K, or MAPK pathways (Figure 3A). Guided by our structure (Figure 1), we generated a panel of IL-21 variants by systematically introducing substitutions to the IL-21–γc interface and testing these variants for modulatory effects on cell signaling by flow cytometry.

$Q116^{IL\text{-}21}$ binds in a pocket of γc formed by the peptide backbone of $P207\text{-}L208\text{-}C209\text{-}G210^{\gamma c}$ and the side chain of $Q127^{IL\text{-}21}$. The side chain Nε of $Q116^{IL\text{-}21}$ forms a hydrogen bond to the backbone carbonyl of $P207^{\gamma c}$, and the Oε makes a solvent-mediated hydrogen bond to the side chain of $Q127^{\gamma c}$. We tested site IIa mutations that we expected to disrupt these interactions. While substitutions at site IIa residues including $D37^{IL\text{-}21}$ and $S113^{IL\text{-}21}$ did not significantly alter pSTAT3 signaling, substitution of the highly conserved residue $Q116^{IL\text{-}21}$ resulted in partial agonism in pSTAT3 and pSTAT1 signaling in the YT-1 human NK cell line, enabling modulation of pSTAT3 E_{max} from 30% to 100% of wild-type (WT) IL-21 (Figures 3B–3D and S3A–S3H). Substitution of $Q116^{IL\text{-}21}$ with small side chains (Ala, Thr) resulted in smaller reductions in E_{max}, potentially by disrupting hydrogen bonds while accommodating solvent in the binding site. Substitution with hydrophobic side chains (Val, Ile) resulted in intermediate E_{max}, potentially by excluding solvent or making potentially unfavorable van der Waals contacts in the γc binding site. Lastly, an acidic residue (Asp) resulted in the most dramatic abolition of pSTAT3 signal, potentially through electrostatic repulsion by the negative dipole of the backbone carbonyls that form the binding pocket without making favorable hydrogen bonds.

We selected a subset of IL-21 partial agonists spanning a range of pSTAT3 E_{max} for further study. We verified that the Q116T and Q116I IL-21 variants exhibited partial agonism in human primary immune cells including $CD4^+$ T cells, $CD8^+$ T cells, $CD19^+$ B cells, and $CD56^+$ NK cells (Figures 3E and S4A–S4F). Across these cell subsets, Q116T elicited a 50%–60% pSTAT3 E_{max} relative to WT, and Q116I elicited a 20%–40% E_{max}, with a slight bias toward $CD8^+$ T cells (Figure 3F). Q116D only elicited a 20% pSTAT3 E_{max} on $CD19^+$ cells, with a 0%–10% pSTAT3 signal induced across all other cell types (Figure 3F).

Tuned IL-21 variants synergize with BCR and CD40 stimulation to activate B cells

Given the role for IL-21 in T-dependent B cell activation, we wished to study the effects of our engineered ligands on B cell activation. In the germinal center, T follicular helper cells (Tfh) provide stimulation to cognate B cells via a T cell receptor (TCR)-pMHC interaction and a CD40L-CD40 interaction (Figure 4A). IL-21 is secreted by Tfh cells and signals on B cells to induce proliferation, class switching, and differentiation of germinal center B cells or plasmablasts.[27] To further understand the role of IL-21 in B cell activation, we tested the effects of our IL-21 variants together with B cell receptor (BCR) and CD40 stimulation on phospho-S6 (pS6) and phospho-ERK (pERK) activation. We found that CD40 and BCR stimulation had additive effects on pERK activation in human B cells, with no added effect of IL-21 stimulation (Figures 4B and S4J). Individually, IL-21 or BCR stimulation each elicited weak activation of pS6, but together, IL-21 and BCR stimulation synergized to induce a strong pS6 signal (Figures 4C and S4G–S4J). Compared with WT IL-21, partial agonists Q116T and Q116I induced undetectable background pS6 activity on B cells in the absence of BCR stimulation (Figures 4C and S4H–S4J). These data highlight the synergistic effect of WT IL-21 and BCR stimulation on pS6 activation and reduced background signaling by the engineered variants.

IL-21 signaling modulates antibody production *in vitro*

Since IL-21 plays an important role in the germinal center reaction, we interrogated the role of IL-21 signaling in human tonsil organoids, an *ex vivo* model of the germinal center reaction.[28] We profiled B cell subsets in human tonsils for expression of IL-21R and γc and found that plasmablasts expressed high levels of IL-21R and γc (Figure 4D). When tonsil organoids were cultured with the addition of IL-21, plasmablasts expressed higher levels of intracellular pS6 (Figure 4E). Plasmablast counts in the tonsil organoid were increased by the addition of WT IL-21, and this increase was modulated when stimulated with Q116 variants (Figure 4F). When stimulated with live attenuated influenza vaccine (LAIV) and IL-21, tonsil organoids from both adult and pediatric donors showed an increase in plasmablast frequencyand immunoglobulin G (IgG) production against influenza (Figures S5B and S5C). Seven days post stimulation with vaccine, we quantified flu-specific IgG produced in the tonsil organoids by conducting ELISAs coated with inactivated influenza vaccine. Tonsil organoids produced higher titers of flu-specific antibody when cultured with WT IL-21 or Q116T (Figures 4G, 4H, and S5D–S5G). WT IL-21 led to the greatest increase in flu-specific antibodies in all five donors (Figure S5G), with Q116T and Q116I leading to stepwise decreases in antibody production corresponding to their relative signaling E_{max} (Figures 4G and 4H). Since these results suggest that IL-21 signaling strength correlates with antibody production, we also explored whether blockade of IL-21 signaling could have the opposite effect. We tested the effects of a single-chain fragment variable (scFv) against IL-21R reported to antagonize IL-21 signaling.[29] Indeed, in tonsil organoids cultured with this scFv

(C) Close-up view comparing helix D of IL-2 (beige) and helix D of IL21 (green), represented as cylinders to show the structural overlap of the site IIa binding residues.

(D) Structure-based sequence alignment between IL-2, IL-4, IL-7, IL-9, and IL-15. Residues contacting γc are highlighted in pink. Residues contacting IL-21R are highlighted in blue.

(E) Close-up views comparing the helix D interaction with γc. A hydrogen bond "hotspot" interaction is highlighted between $P207^{\gamma c}$ (shown in pink) and $Q116^{IL\text{-}21}$ (dark green), $Q126^{IL\text{-}2}$ (beige), $R121^{IL\text{-}4}$ (purple), and $Q108^{IL\text{-}15}$ (light green).

(F) Close-up views comparing the helix B interaction with γc. Hydrogen bonding is highlighted between $T105^{\gamma c}$ (shown in pink) and $D37^{IL\text{-}21}$ (dark green) or $D30^{IL\text{-}15}$ (light green). There is no hydrogen bonding between γc and helix B for either IL-2 (beige) or IL-4 (purple).

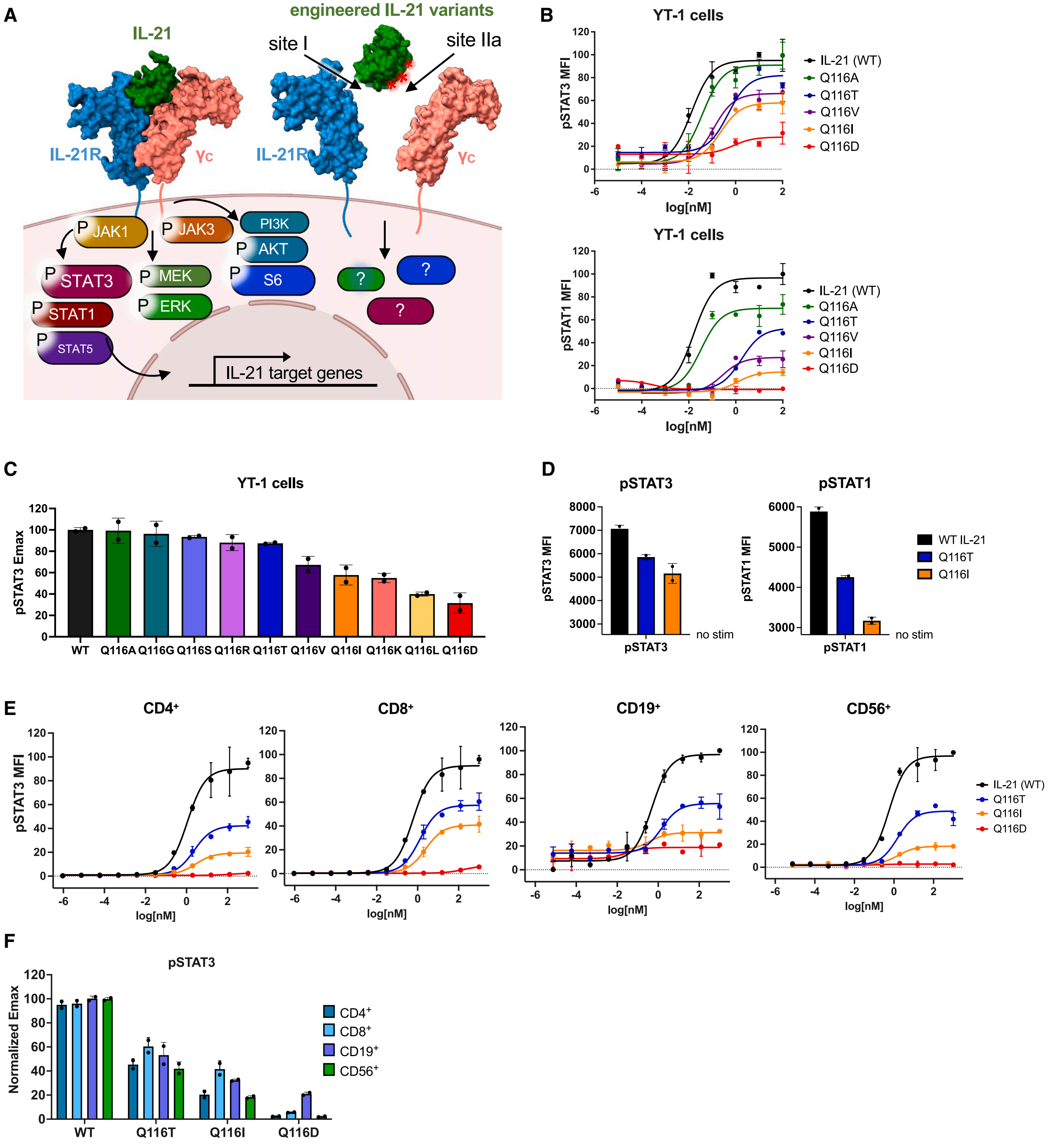

Figure 3. Engineering the IL-21–γc interface modulates downstream signaling pathways

(A) Schematic of the signaling pathways activated by WT IL-21 and the design of IL-21 variants with substitutions along the IL-21–γc interface.

(B) Dose-response curves for phopsho-STAT3 (top) and phospho-STAT1 (bottom) in YT-1 cells stimulated with WT IL-21 or the indicated variants for 20 min and analyzed by flow cytometry. Data are mean ± SD for two replicates, shown as percentage of maximal WT IL-21 MFI.

(C) Normalized E_{max} values for phopsho-STAT3, calculated from the dose-response curves shown in (B). Data are mean ± SD for two replicates.

(legend continued on next page)

against IL-21R, we observed an abolished antibody response to flu (Figure 4I). These results suggest that in the tonsil organoid model IL-21 signaling is essential for the antibody response generated post vaccination, and IL-21 analogs can stimulate graded increases in antibody production.

Given the reported role of IL-21 in autoimmunity, we also assessed whether our IL-21 partial agonists reduced autoantibody production relative to WT. In tonsils stimulated with IL-21 or IL-21 variants, we conducted ELISAs to detect IgG produced against common self-antigens including double-stranded DNA (dsDNA), DNA-repair protein Ku, Sjögren's syndrome antigen B (La/SS-B), a small nuclear ribonucleoprotein (SNRNP70), and insulin. IL-21 increased the production of IgM against dsDNA and Ku, as well as the production of IgG against La (Figures S6A and S6B). Compared with WT IL-21, partial agonists Q116T and Q116I diminished the production of all autoantibodies to varied extents (Figures S6A and S6B). These results suggest that a downtuned variant of IL-21 such as Q116T may retain some of the beneficial adjuvant activity of IL-21 while reducing the potential induction of self-reactive antibodies.

DISCUSSION

Structure-based design of IL-21 partial agonists

Common-gamma-chain cytokines are critical mediators of adaptive immunity and are therapeutic targets for both agonism and antagonism. One of the principal barriers for therapeutic use of γc cytokines is their pleiotropy. Structural information on γc cytokine receptor complexes has enabled engineering of γc cytokines to reduce pleiotropy and modulate their actions in order to enhance clinical efficacy as well as safety. For example, structures of IL-2 have unlocked the engineering of IL-2 therapeutics.[26,30–32] IL-21 is a key γc cytokine with a broad range of activities on B and T cells, but its pleiotropy hinders its clinical use, for example in cancer, by virtue of its activity on CD8$^+$ T cells or as a vaccine adjuvant, by virtue of its activity on Tfh and B cells.[1,33] Our study presents structures of the IL-21 receptor complex with IL-21R and γc, extending the menu of γc cytokine receptor complexes available for engineering.

A structural comparison of IL-21 with the structures of IL-2, IL-4, and IL-15 revealed the mechanisms of receptor sharing across the γc family of cytokines. Particularly interesting is the identification of a "hotspot" residue on IL-21 helix D at Q116 that makes hydrogen bonds with the γc backbone and is highly conserved across the γc family of cytokines. Our structural studies also revealed an unexpected hexameric assembly suggested by both crystallography and cryo-EM. The 2:2:2 complex is bridged by a homodimeric IL-21R–IL-21R interface which, to our knowledge, has not been observed in the structures of other γc family cytokines. The higher-order stoichiometry of the IL-21 receptor complex is reminiscent of the 2:2:2 stoichiometry observed in the IL-6–IL-6Rα–gp130 complex[34] and the common-beta (βc) family of cytokines.[35–37] Since IL-21 makes no contact with the 21R from the adjacent 21-21R complex, the hexamer is mediated largely by receptor-receptor contacts. Although questions remain about the physiological significance of the hexamer and whether IL-21R dimerization contributes to cooperative receptor assembly or the alteration of intracellular signaling cascades, the persistence of the hexamer by multiple structural approaches suggests that the entity could form on cell surfaces.

IL-21 analogs drive a range of B cell activation

We show that modulation of γc heterodimerization efficiency with IL-21R through rationally designed amino acid substitutions in site IIa can result in rheostat-like signaling output with cell-type-specific effects. For example, by tuning the affinity of the IL-21–γc interface, we were able to alter downstream IL-21 signaling through the pAKT/pS6 and STAT pathways. Substitutions at residue Q116^{IL-21} resulted in IL-21 variants with different degrees of biased signaling, with larger reductions in pSTAT1 signaling relative to pSTAT3.

This structure-based engineering approach enabled us to interrogate IL-21 biology in T-dependent B cell activation. In human B cells, we observed synergy between BCR ligation and IL-21 on activation of the pAKT/pS6 pathway. These results are consistent with literature describing IL-21 as an "executor of B cell fate" that is capable of inducing apoptosis or proliferation of B cells depending on other cues and that can collaborate with CD40 stimulation or BCR crosslinking to drive B cell proliferation.[38–40] Similarly, a recent study suggests that IL-21 synergizes with BCR and CD40 stimulation by lowering the BCR affinity threshold for T-dependent B cell activation.[41] These prior studies support a model for T-dependent B cell activation that integrates three signals: (1) BCR ligation, (2) CD40 co-stimulation by the T cell, and (3) IL-21 (Figure 4A). This model is highly analogous to that for antigen-specific T cell activation that requires TCR stimulation, CD28 co-stimulation, and IL-2.[42]

Effects of IL-21 on humoral immunity

Our engineered ligands enabled us to interrogate the role of IL-21 signaling on antibody production in human tonsil organoids. Since tonsils are a secondary lymphoid organ, these organoid experiments enabled us to gain insight into human physiology. These studies showed that IL-21 can potentiate the germinal center reaction, stimulate plasmablast proliferation, and enhance flu-specific antibody responses. These results clarify the critical role of IL-21 signaling for antibody production in human tonsil organoids. These findings align with recent findings showing that IL-21 determines the magnitude of the germinal center reaction and supports plasma cell differentiation in mice.[4]

(D) Maximum MFI of phospho-STAT3 or phospho-STAT1 in YT-1 cells treated with saturating concentrations of IL-21 or variant. Data are mean ± SD for two replicates. The y axis is scaled to begin at the unstimulated background.

(E) Dose-response curves for phospho-STAT3 in human CD4$^+$ T cells, CD8$^+$ T cells, CD19$^+$ B cells, and CD56$^+$ NK cells. Cells were stimulated with WT IL-21 or the indicated variants for 20 min and analyzed by flow cytometry. Data are mean ± SD for two replicates, shown as percentage of maximal WT IL-21 MFI.

(F) Normalized E_{max} values for phospho-STAT3 in CD4$^+$, CD8$^+$, CD19$^+$, and CD56$^+$ human PBMCs calculated from dose-response curves shown in (E). Data are mean ± SD for two replicates.

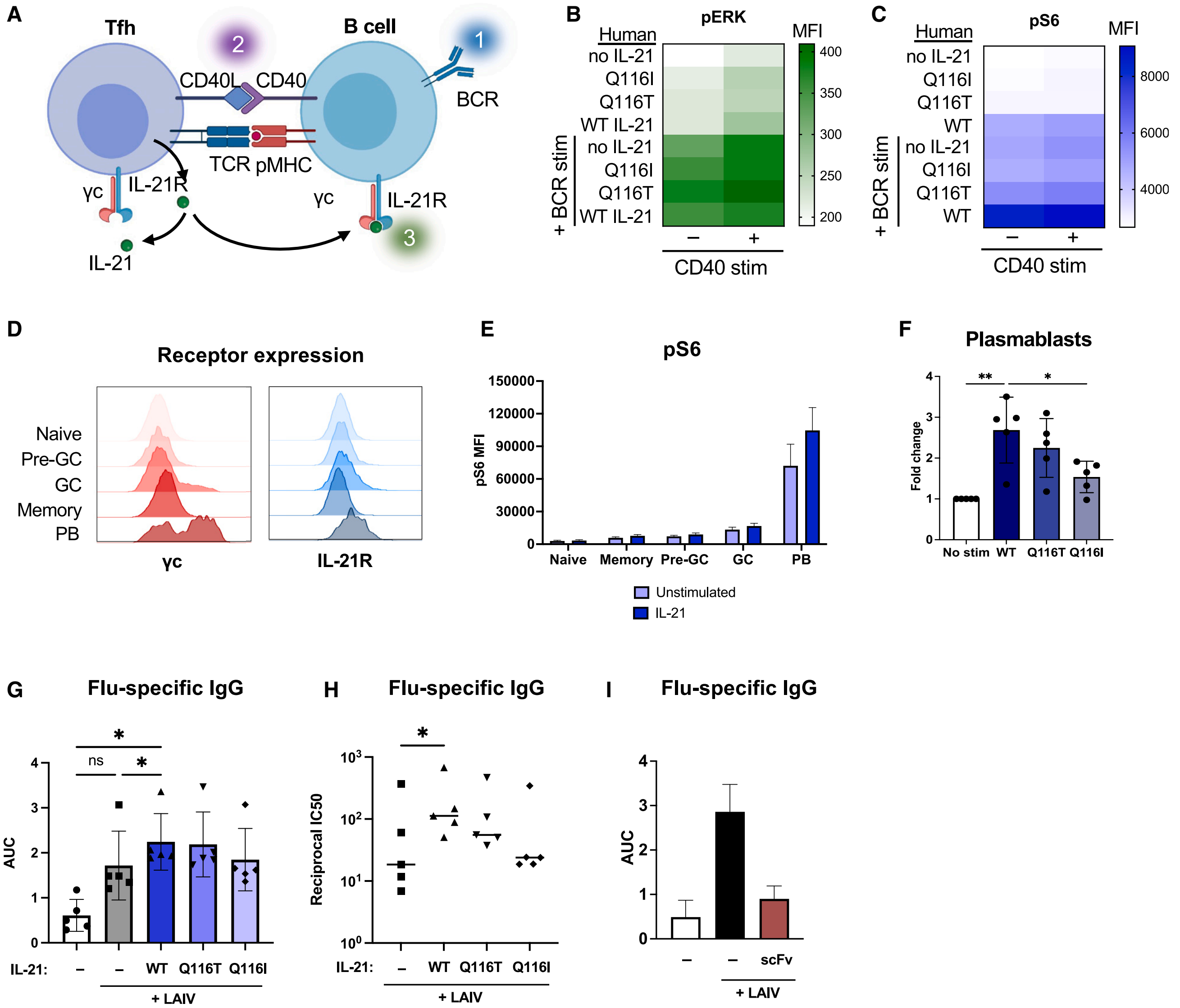

Figure 4. IL-21 variants modulate B cell activation and antibody production

(A) Schematic depicting the T-dependent B cell interaction between a T follicular helper cell (Tfh) and B cell.

(B) Activation of pERK in human B cells stimulated with anti-CD40, BCR crosslinking, and WT IL-21 or indicated variant. Data shown are the MFI analyzed by flow cytometry.

(C) Activation of pS6 in human B cells stimulated with anti-CD40, BCR crosslinking, and WT IL-21 or indicated variant. Data shown are the MFI analyzed by flow cytometry.

(D) Representative histograms of γc and IL-21R surface expression of naive, pre-germinal center (pre-GC), germinal center (GC), memory, and plasmablast (PB) B cells from unstimulated human tonsil organoids.

(E) Activation of intracellular pS6 analyzed by flow cytometry in B cell subsets in tonsil organoids cultured with or without 100 nM WT IL-21. Data are mean ± SD for five human donors.

(F) Frequency of plasmablasts from tonsil organoids cultured for 7 days in the presence of 100 nM IL-21 or variant, normalized to unstimulated tonsils. Data are mean ± SD for five human donors. *$p \leq 0.05$, **$p \leq 0.01$ by one-way ANOVA.

(G and H) Flu-specific IgG quantified by ELISA from the tonsil organoids vaccinated with LAIV and indicated IL-21 variant on day 7 post stimulation with vaccine. Data were fit by non-linear regression, and area under the curve (AUC) and IC_{50}s were calculated in Prism. Data are mean AUC ± SD (G) or $\log(1/IC_{50})$ (H) for five human donors. *$p \leq 0.05$ by one-way ANOVA; ns, not significant.

(I) Flu-specific IgG detected by ELISA on day 7 post vaccination from tonsil organoids cultured with or without an IL-21R blocking scFv. Data are mean ± SD for two human donors.

Previously, a major barrier to leveraging IL-21 therapeutically was the possibility of inducing autoimmune disease. Evident in the literature and in our experiments, IL-21 can drive the non-specific production of lupus-like antibodies in the absence of BCR ligation and co-stimulation.[12] This demonstrates the need for downtuned IL-21 variants that activate moderate-affinity B cells without activating autoreactive B cells. Broadly, the structure-based engineering of downtuned cytokines is a well-established approach of narrowing the pleiotropic effects of cytokines for targeted therapeutic use. For example, IL-12 partial agonists facilitated tumor clearance *in vivo* with reduced NK cell-mediated toxicities, and IL-10 partial agonists displayed myeloid cell-biased immunosuppression without T cell-mediated inflammation.[23,24] In the case of IL-21, our engineered Q116T variant enhanced the production of flu-specific antibodies while reducing the production of non-specific autoantibodies. Our downtuned variants exhibited decreased pS6 activation in B cells and reduced autoantibody production in human tonsil organoids, proportional to signaling E_{max}. These insights into IL-21 signaling and engineering could guide future work to explore the use and tuning of IL-21 as a potential vaccine adjuvant.

Concluding remarks

We determine the structural mechanism by which IL-21 signaling is linked to downstream pS6 and pSTAT activation. In human tonsil organoids, we show that IL-21 receptor agonists can augment antibody production by B cells. By tuning IL-21 signaling at the IL-21–γc interface, we generated IL-21 variants that modulate levels of B cell activation and antibody production. These findings clarify the structural mechanisms of IL-21 signaling and may guide further studies of IL-21 as a potential vaccine adjuvant or for other clinical uses.

Limitations of the study

In this study, we observed a 2:2:2 assembly of the IL-21 receptor complex by cryo-EM and crystallography in addition to the expected 1:1:1 ternary complex. The physiological relevance of the 2:2:2 complex remains to be determined. Although the determination of the structure by two independent structural techniques suggests that the complex could form on cells, it is still possible that the 2:2:2 complex is an artifact that occurs only with recombinant proteins. Secondly, we engineered human IL-21 variants based on our structure of the human IL-21 receptor complex. Since we were working with human molecules, we tested the effects of these variants on antibody production in a human tonsil organoid. We observed that IL-21 partial agonism induced a range of flu-specific and non-specific antibody production upon vaccination. However, the clinical feasibility of using IL-21 variants as vaccine adjuvants must be further tested *in vivo*. Longer term vaccination experiments will be needed to determine whether IL-21 engineering can enhance the titers, breadth, or durability of antibody production post vaccination.

STAR★METHODS

Detailed methods are provided in the online version of this paper and include the following:

- KEY RESOURCES TABLE
- RESOURCE AVAILABILITY
  - Lead contact
  - Materials availability
  - Data and code availability
- EXPERIMENTAL MODEL AND SUBJECT DETAILS
  - Mammalian cell lines and culture conditions
  - Insect cell lines and culture conditions
  - Human primary cells and culture conditions
  - Human tonsil organoid culture
- METHOD DETAILS
  - Protein production and purification
  - Crystallization, data collection, and refinement
  - Cryo-electron microscopy
  - Cryo-EM image processing and data processing
  - Signaling assays in YT-1 cells
  - Signaling assays in human PBMCs
  - Tonsil organoid culture
  - Antibody detection by ELISA
  - Human B cell activation assays
- QUANTIFICATION AND STATISTICAL ANALYSIS

SUPPLEMENTAL INFORMATION

Supplemental information can be found online at https://doi.org/10.1016/j.celrep.2023.112657.

ACKNOWLEDGMENTS

The authors would like to thank Peter Kim, Caleb Glassman, Marta Borowska, Steven Wilson, Harrison Besser, Camilo Espinosa Bernal, and all members of the Garcia lab for helpful feedback and discussion. We thank Liz Montabana and the Stanford cEMc facility for cryo-EM microscope access. K.C.G. and M.M.D. are investigators with the Howard Hughes Medical Institute. G.C.A. is supported by the Hertz Foundation Fellowship. N.A.C. is supported by a CIHR postdoctoral fellowship. G.C.A. is supported by Stanford University Medical Scientist Training Program grant T32-GM007365 and a Hertz Fellowship. T.U.J.B. is supported by the Knight-Hennessy Graduate Scholarship Fund and a CIHR Doctoral Foreign Study Award (FRN:170770). This work was supported by the Bill and Melinda Gates Foundation (K.C.G.), National Institutes of Health (NIH) RO1-AI51321 (K.C.G.), and 5U19AI057229-18 (M.M.D.). Use of SSRL at Stanford Linear Accelerator Center National Accelerator Laboratory is supported by the US Department of Energy Office of Science, Office of Basic Energy Sciences under contract DE-AC02-76SF00515. The SSRL Structural Molecular Biology Program is supported by the Department of Energy, Office of Biological and Environmental Research, and the National Institutes of Health, National Institute of General Medical Sciences (including P41GM103393). Figures 3A and 4A were made using BioRender.

AUTHOR CONTRIBUTIONS

G.C.A. and K.C.G. formulated the research plan and interpreted results. G.C.A., K.M.J., N.A.C., T.U.J.B., S.T., R.A.S., Q.Y., and L.L.S. designed and performed experiments and analyzed the data. M.M.D. supervised the tonsil organoid experiments. G.C.A. and K.C.G. wrote the manuscript with input from all authors.

DECLARATION OF INTERESTS

The authors declare no competing interests.

INCLUSION AND DIVERSITY

We support inclusive, diverse, and equitable conduct of research.

Received: January 2, 2023
Revised: May 12, 2023
Accepted: June 1, 2023
Published: June 19, 2023

REFERENCES

1. Spolski, R., and Leonard, W.J. (2014). Interleukin-21: a double-edged sword with therapeutic potential. Nat. Rev. Drug Discov. *13*, 379–395. https://doi.org/10.1038/nrd4296.
2. Stolfi, C., Pallone, F., Macdonald, T.T., and Monteleone, G. (2012). Interleukin-21 in cancer immunotherapy: {Friend} or foe? Oncoimmunology *1*, 351–354.
3. Linterman, M.A., Beaton, L., Yu, D., Ramiscal, R.R., Srivastava, M., Hogan, J.J., Verma, N.K., Smyth, M.J., Rigby, R.J., and Vinuesa, C.G. (2010). IL-21 acts directly on B cells to regulate Bcl-6 expression and germinal center responses. J. Exp. Med. *207*, 353–363. https://doi.org/10.1084/jem.20091738.
4. Quast, I., Dvorscek, A.R., Pattaroni, C., Robinson, M.J., Zotos, D., Tarlinton, D.M., Quast, I., Dvorscek, A.R., Pattaroni, C., Steiner, T.M., et al. (2022). Article Interleukin-21, acting beyond the immunological synapse, independently controls T follicular helper and germinal center B cells Article Interleukin-21, acting beyond the immunological synapse, independently controls T follicular helper and germi. Immunity *55*, 1414–1430.e5. https://doi.org/10.1016/j.immuni.2022.06.020.
5. Tangye, S.G., and Ma, C.S. (2020). Regulation of the germinal center and humoral immunity by interleukin-21. J. Exp. Med. *217*, e20191638. https://doi.org/10.1084/jem.20191638.
6. Kuchen, S., Robbins, R., Sims, G.P., Sheng, C., Phillips, T.M., Lipsky, P.E., and Ettinger, R. (2007). Essential role of IL-21 in B cell activation, expansion, and plasma cell generation during CD4 + T cell-B cell collaboration. J. Immunol. *179*, 5886–5896. https://doi.org/10.4049/jimmunol.179.9.5886.
7. Croce, M., Rigo, V., and Ferrini, S. (2015). IL-21: a pleiotropic cytokine with potential applications in Oncology. J. Immunol. Res. *2015*, 696578. https://doi.org/10.1155/2015/696578.
8. Bolesta, E., Kowalczyk, A., Wierzbicki, A., Eppolito, C., Kaneko, Y., Takiguchi, M., Stamatatos, L., Shrikant, P.A., and Kozbor, D. (2006). Increased level and longevity of protective immune responses induced by DNA vaccine expressing the HIV-1 Env glycoprotein when combined with IL-21 and IL-15 gene delivery. J. Immunol. *177*, 177–191.
9. Andersson, A.K., Feldmann, M., and Brennan, F.M. (2008). Neutralizing IL-21 and IL-15 inhibits pro-inflammatory cytokine production in rheumatoid arthritis. Scand. J. Immunol. *68*, 103–111. https://doi.org/10.1111/j.1365-3083.2008.02118.x.
10. Clough, L.E., Wang, C.J., Schmidt, E.M., Booth, G., Hou, T.Z., Ryan, G.A., and Walker, L.S.K. (2008). Release from regulatory T cell-mediated suppression during the onset of tissue-specific autoimmunity is associated with elevated IL-21. J. Immunol. *180*, 5393–5401. https://doi.org/10.4049/jimmunol.180.8.5393.
11. Sawalha, A.H., Kaufman, K.M., Kelly, J.A., Adler, A.J., Aberle, T., Kilpatrick, J., Wakeland, E.K., Li, Q.Z., Wandstrat, A.E., Karp, D.R., et al. (2008). Genetic association of interleukin-21 polymorphisms with systemic lupus erythematosus. Ann. Rheum. Dis. *67*, 458–461. https://doi.org/10.1136/ard.2007.075424.
12. Herber, D., Brown, T.P., Liang, S., Young, D.A., Collins, M., and Dunussi-Joannopoulos, K. (2007). IL-21 has a pathogenic role in a lupus-prone mouse model and its blockade with IL-21R.Fc reduces disease progression. J. Immunol. *178*, 3822–3830.
13. Wang, S., Wang, J., Kumar, V., Karnell, J.L., Naiman, B., Gross, P.S., Rahman, S., Zerrouki, K., Hanna, R., Morehouse, C., et al. (2018). IL -21 drives expansion and plasma cell differentiation of autoreactive CD11c hi T-bet+ B cells in SLE. Nat. Commun. *9*, 1758.
14. Wei, L., Laurence, A., Elias, K.M., and O'Shea, J.J. (2007). IL-21 is produced by Th17 cells and drives IL-17 production in a STAT3-dependent manner. J. Biol. Chem. *282*, 34605–34610. https://doi.org/10.1074/jbc.M705100200.
15. Fina, D., Sarra, M., Fantini, M.C., Rizzo, A., Caruso, R., Caprioli, F., Stolfi, C., Cardolini, I., Dottori, M., Boirivant, M., et al. (2008). Regulation of gut inflammation and Th17 cell response by interleukin-21. Gastroenterology *134*, 1038–1048. https://doi.org/10.1053/j.gastro.2008.01.041.
16. Leonard, W.J., and Wan, C.K. (2016). IL-21 signaling in immunity. F1000Res. *5*, F1000 Faculty Rev-224. https://doi.org/10.12688/f1000research.7634.1.
17. Hamming, O.J., Kang, L., Svensson, A., Karlsen, J.L., Rahbek-Nielsen, H., Paludan, S.R., Hjorth, S.A., Bondensgaard, K., and Hartmann, R. (2012). Crystal structure of interleukin-21 receptor (IL-21R) bound to IL-21 reveals that sugar chain interacting with WSXWS motif is integral part of IL-21R. J. Biol. Chem. *287*, 9454–9460. https://doi.org/10.1074/jbc.M111.311084.
18. Bondensgaard, K., Breinholt, J., Madsen, D., Omkvist, D.H., Kang, L., Worsaae, A., Becker, P., Schiødt, C.B., and Hjorth, S.A. (2007). The existence of multiple conformers of interleukin-21 directs engineering of a superpotent analogue. J. Biol. Chem. *282*, 23326–23336. https://doi.org/10.1074/jbc.M701313200.
19. Wang, X., Lupardus, P., LaPorte, S.L., and Garcia, K.C. (2009). Structural biology of shared cytokine receptors. Annu. Rev. Immunol. *27*, 29–60.
20. Wang, X., Rickert, M., and Garcia, K.C. (2005). Structure of the quaternary complex of interleukin-2 with its α, ß, and γc receptors. Science *310*, 1159–1163.
21. LaPorte, S.L., Juo, Z.S., Vaclavikova, J., Colf, L.A., Qi, X., Heller, N.M., Keegan, A.D., and Garcia, K.C. (2008). Molecular and structural basis of cytokine receptor pleiotropy in the interleukin-4/13 system. Cell *132*, 259–272. https://doi.org/10.1016/j.cell.2007.12.030.
22. Ring, A.M., Lin, J.X., Feng, D., Mitra, S., Rickert, M., Bowman, G.R., Pande, V.S., Li, P., Moraga, I., Spolski, R., et al. (2012). Mechanistic and structural insight into the functional dichotomy between IL-2 and IL-15. Nat. Immunol. *13*, 1187–1195. https://doi.org/10.1038/ni.2449.
23. Saxton, R.A., Tsutsumi, N., Su, L.L., Abhiraman, G.C., Mohan, K., Henneberg, L.T., Aduri, N.G., Gati, C., and Garcia, K.C. (2021). Structure-based decoupling of the pro-and anti-inflammatory functions of interleukin-10. Science *371*, eabc8433.
24. Glassman, C.R., Mathiharan, Y.K., Jude, K.M., Su, L., Panova, O., Lupardus, P.J., Spangler, J.B., Ely, L.K., Thomas, C., Skiniotis, G., et al. (2021). Structural basis for IL-12 and IL-23 receptor sharing reveals a gateway for shaping actions on T versus NK cells. Cell *184*, 983–999.e24. https://doi.org/10.1016/j.cell.2021.01.018.
25. Saxton, R.A., Henneberg, L.T., Calafiore, M., Su, L., Jude, K.M., Hanash, A.M., and Garcia, K.C. (2021). The tissue protective functions of interleukin-22 can be decoupled from pro-inflammatory actions through structure-based design. Immunity *54*, 660–672.e9. https://doi.org/10.1016/j.immuni.2021.03.008.
26. Glassman, C.R., Su, L., Majri-Morrison, S.S., Winkelmann, H., Mo, F., Li, P., Pérez-Cruz, M., Ho, P.P., Koliesnik, I., Nagy, N., et al. (2021). Calibration of cell-intrinsic interleukin-2 response thresholds guides design of a regulatory T cell biased agonist. Elife *10*, e65777. https://doi.org/10.7554/eLife.65777.
27. Pulendran, B., S Arunachalam, P., O'Hagan, D.T., and O'Hagan, D.T. (2021). Emerging concepts in the science of vaccine adjuvants. Nat. Rev. Drug Discov. *20*, 454–475. https://doi.org/10.1038/s41573-021-00163-y.
28. Wagar, L.E., Salahudeen, A., Constantz, C.M., Wendel, B.S., Lyons, M.M., Mallajosyula, V., Jatt, L.P., Adamska, J.Z., Blum, L.K., Gupta, N., et al.

(2021). Modeling human adaptive immune responses with tonsil organoids. Nat. Med. *27*, 125–135. https://doi.org/10.1038/s41591-020-01145-0.

29. Young, D., Whitters, M.J., Valge-Archer, V., Collins, M., Williams, A.J., and Witek, J. (2004). WO 2004/083249A2. https://patentscope.wipo.int/search/en/detail.jsf?docId=WO2004083249&_cid=P22-LIWCMY-41014-5

30. Hernandez, R., Põder, J., LaPorte, K.M., and Malek, T.R. (2022). Engineering IL-2 for immunotherapy of autoimmunity and cancer. Nat. Rev. Immunol. *22*, 614–628. https://doi.org/10.1038/s41577-022-00680-w.

31. Sockolosky, J.T., Trotta, E., Parisi, G., Picton, L., Su, L.L., Le, A.C., Chhabra, A., Silveria, S.L., George, B.M., King, I.C., et al. (2018). Selective targeting of engineered T cells using orthogonal IL-2 cytokine-receptor complexes. Science *359*, 1037–1042. https://doi.org/10.1126/science.aar3246.

32. Zhang, Q., Hresko, M.E., Picton, L.K., Su, L., Hollander, M.J., Nunez-Cruz, S., Zhang, Z., Assenmacher, C.A., Sockolosky, J.T., Garcia, K.C., et al. (2021). A human orthogonal IL-2 and IL-2Rβ system enhances CAR T cell expansion and antitumor activity in a murine model of leukemia. Sci. Transl. Med. *13*, eabg6986. https://doi.org/10.1126/scitranslmed.abg6986.

33. Spolski, R., and Leonard, W.J. (2008). Interleukin-21: Basic biology and implications for cancer and autoimmunity. Annu. Rev. Immunol. *26*, 57–79. https://doi.org/10.1146/annurev.immunol.26.021607.090316.

34. Boulanger, M.J., Chow, D.c., Brevnova, E.E., and Garcia, K.C. (2003). Hexameric structure and assembly of the interleukin-6/IL-6 α-receptor/gp130 complex. Science *300*, 2101–2104. https://doi.org/10.1126/science.1083901.

35. Hansen, G., Hercus, T.R., McClure, B.J., Stomski, F.C., Dottore, M., Powell, J., Ramshaw, H., Woodcock, J.M., Xu, Y., Guthridge, M., et al. (2008). The structure of the GM-CSF receptor complex reveals a distinct mode of cytokine receptor activation. Cell *134*, 496–507. https://doi.org/10.1016/j.cell.2008.05.053.

36. Carr, P.D., Gustin, S.E., Church, A.P., Murphy, J.M., Ford, S.C., Mann, D.A., Woltring, D.M., Walker, I., Ollis, D.L., and Young, I.G. (2001). Structure of the complete extracellular domain of the common β subunit of the human GM-CSF, IL-3, and IL-5 receptors reveals a novel dimer configuration. Cell *104*, 291–300. https://doi.org/10.1016/S0092-8674(01)00213-6.

37. Broughton, S.E., Hercus, T.R., Nero, T.L., Dottore, M., McClure, B.J., Dhagat, U., Taing, H., Gorman, M.A., King-Scott, J., Lopez, A.F., et al. (2016). Conformational changes in the GM-CSF receptor suggest a molecular mechanism for affinity conversion and receptor signaling. Structure *24*, 1271–1281. https://doi.org/10.1016/j.str.2016.05.017.

38. Konforte, D., Simard, N., and Paige, C.J. (2009). IL-21: an executor of B cell fate. J. Immunol. *182*, 1781–1787. https://doi.org/10.4049/jimmunol.0803009.

39. Good, K.L., Bryant, V.L., and Tangye, S.G. (2006). Kinetics of human B cell behavior and amplification of proliferative responses following stimulation with IL-21. J. Immunol. *177*, 5236–5247. https://doi.org/10.4049/jimmunol.177.8.5236.

40. Ettinger, R., Sims, G.P., Fairhurst, A.-M., Robbins, R., da Silva, Y.S., Spolski, R., Leonard, W.J., and Lipsky, P.E. (2005). IL-21 induces differentiation of human naive and memory B cells into antibody-secreting plasma cells. J. Immunol. *175*, 7867–7879. https://doi.org/10.4049/jimmunol.175.12.7867.

41. Dvorscek, A.R., McKenzie, C.I., Robinson, M.J., Ding, Z., Pitt, C., O'Donnell, K., Zotos, D., Brink, R., Tarlinton, D.M., and Quast, I. (2021). IL-21 lowers the B cell receptor affinity threshold for participation in a T cell dependent immune response. Preprint at bioRxiv. https://doi.org/10.1101/2022.01.21.476732.

42. Ledbetter, J.A., Imboden, J.B., Schieven, G.L., Grosmaire, L.S., Rabinovitch, P.S., Lindsten, T., Thompson, C.B., and June, C.H. (1990). CD28 ligation in T-cell activation: evidence for two signal transduction pathways. Blood *75*, 1531–1539. https://doi.org/10.1182/blood.v75.7.1531.bloodjournal7571531.

43. Liebschner, D., Afonine, P.V., Baker, M.L., Bunkóczi, G., Chen, V.B., Croll, T.I., Hintze, B., Hung, L.W., Jain, S., McCoy, A.J., et al. (2019). Macromolecular structure determination using X-rays, neutrons and electrons: recent developments in Phenix. Acta Crystallogr. D Struct. Biol. *75*, 861–877. https://doi.org/10.1107/S2059798319011471.

44. Emsley, P., Lohkamp, B., Scott, W.G., and Cowtan, K. (2010). Features and development of Coot. Acta Crystallogr. D Biol. Crystallogr. *66*, 486–501. https://doi.org/10.1107/S0907444910007493.

45. Goddard, T.D., Huang, C.C., Meng, E.C., Pettersen, E.F., Couch, G.S., Morris, J.H., and Ferrin, T.E. (2018). UCSF ChimeraX: meeting modern challenges in visualization and analysis. Protein Sci. *27*, 14–25. https://doi.org/10.1002/pro.3235.

46. Krissinel, E., and Henrick, K. (2007). Inference of macromolecular assemblies from crystalline state. J. Mol. Biol. *372*, 774–797. https://doi.org/10.1016/j.jmb.2007.05.022.

47. Morin, A., Eisenbraun, B., Key, J., Sanschagrin, P.C., Timony, M.A., Ottaviano, M., and Sliz, P. (2013). Collaboration gets the most out of software. Elife *2*, e01456. https://doi.org/10.7554/eLife.01456.

48. Mastronarde, D.N. (2005). Automated electron microscope tomography using robust prediction of specimen movements. J. Struct. Biol. *152*, 36–51. https://doi.org/10.1016/j.jsb.2005.07.007.

49. Punjani, A., Rubinstein, J.L., Fleet, D.J., and Brubaker, M.A. (2017). CryoSPARC: algorithms for rapid unsupervised cryo-EM structure determination. Nat. Methods *14*, 290–296. https://doi.org/10.1038/nmeth.4169.

50. Kabsch, W. (2010). {\it XDS}. Acta Crystallogr. Sect. D *66*, 125–132. https://doi.org/10.1107/S0907444909047337.

51. McCoy, A.J., Grosse-Kunstleve, R.W., Adams, P.D., Winn, M.D., Storoni, L.C., and Read, R.J. (2007). Phaser crystallographic software. J. Appl. Crystallogr. *40*, 658–674. https://doi.org/10.1107/S0021889807021206.

52. Kallewaard, N.L., Corti, D., Collins, P.J., Neu, U., McAuliffe, J.M., Benjamin, E., Wachter-Rosati, L., Palmer-Hill, F.J., Yuan, A.Q., Walker, P.A., et al. (2016). Structure and function analysis of an antibody recognizing all influenza A subtypes. Cell *166*, 596–608. https://doi.org/10.1016/j.cell.2016.05.073.

STAR★METHODS

KEY RESOURCES TABLE

REAGENT or RESOURCE	SOURCE	IDENTIFIER
**Antibodies**		
Anti-human CD69 PE	BioLegend	Cat#310906; RRID:AB_314841
Anti-human CD4 Pacific Blue	BioLegend	Cat#300521; RRID:AB_493098
Anti-Stat3 (pY705) Alexa Fluor® 647	BD	Cat#557815; RRID:AB_647144
Anti-Stat1 (pY701) Alexa Fluor® 488	BD	Cat#612596; RRID:AB_399879
Anti-human CD8 Brilliant Violet 605	BioLegend	Cat#344742; RRID:AB_2566513
Human TruStain FcX™ (Fc Receptor Blocking Solution)	BioLegend	Cat#422302; RRID:AB_2818986
Anti-human CD3 clone UCHT1, RUO Pacific Blue™	BioLegend	Cat#300431; RRID:AB_1595437
Anti-human CD27 PE/Cyanine7	BioLegend	Cat#302837; RRID:AB_2561918
Anti-human CD3 APC/Cy7	BioLegend	Cat#300318; RRID:AB_314054
Anti-human CXCR5 FITC	BioLegend	Cat#356914; RRID:AB_2561896
Anti-human CD19 PerCP/Cyanine5.5	BioLegend	Cat#302230; RRID:AB_2073119
Anti-human CD38 APC	BioLegend	Cat#303510; RRID:AB_314362
Anti-human CD45 Brilliant Violet 605	BioLegend	Cat#304042; RRID:AB_2562106
Anti-human PD1 Brilliant Violet 785	BioLegend	Cat#329930; RRID:AB_2563443
Goat anti-human IgG-HRP	Southern Biotech	Cat#2040-05; RRID:AB_2795644
Goat anti-human IgM-HRP	Southern Biotech	Cat#2020-05; RRID:AB_2795603
Goat anti-human Ig-HRP	Southern Biotech	Cat#2010-05; RRID:AB_2795564
Mouse anti-human Lambda	Southern Biotech	Cat#9180-01; RRID:AB_2796674
Mouse anti-human Kappa	Southern Biotech	Cat#9230-01; RRID:AB_2796705
Anti-human IL-21 Brilliant Violet 421	BD Biosciences	Cat#564755; RRID:AB_2738933
Anti-Stat5 (pY694) Alexa Fluor® 488	BD Biosciences	Cat#612598; RRID:AB_399881
Anti- phospho Akt (Ser473) (D9E) XP® Rabbit mAb (Alexa Fluor® 647 Conjugate)	Cell Signaling Technologies	Cat#4075S; RRID:AB_916029
Anti-phospho-S6 Ribosomal Protein (Ser235/236) (D57.2.2E) XP® Rabbit mAb (Alexa Fluor® 647 Conjugate)	Cell Signaling Technologies	Cat#4851S; RRID:AB_10695457
Ultra-LEAF™ Purified anti-human CD40 Antibody	BioLegend	Cat#668103; RRID:AB_2814510
Goat Anti-Human Lambda-BIOT	Southern Biotech	Cat#2070-08; RRID:AB_2795755
Goat Anti-Human Kappa-BIOT	Southern Biotech	Cat#2060-08; RRID:AB_2795723
**Bacterial and virus strains**		
Mix & Go Competent Cells – DH5α	Zymo Research	Cat#T3007
**Chemicals, peptides, and recombinant proteins**		
Endo Hf	NEB	Cat#P0703S
Diatomaceous earth	Sigma	Cat#D3877
Penicillin-Streptomycin	Gibco	Cat#15-140-163
NheI-HF	NEB	Cat#R3131L
1-Step™ Ultra TMB-ELISA Substrate Solution	Thermo Fisher	Cat#34028
Recombinant Human BAFF	BioLegend	Cat#559606
MilliporeSigma™ Millicell™ Culture Plate Inserts	Fisher Scientific	Cat#PICM01250
Normocin	Fisher Scientific	Cat#NC9273499
Insulin-Transferrin-Selenium (ITS -G)	Thermo Fisher	Cat#41400045
Deoxyribonucleic acid from human placenta	Sigma	Cat#D3035
Carboxypeptidase A	Sigma	Cat#C9268
Carboxypeptidase B	Sigma	Cat#217356
Phosphate Buffered Saline (PBS)	Gibco	Cat#20012-050

(*Continued on next page*)

Continued

REAGENT or RESOURCE	SOURCE	IDENTIFIER
Fetal Bovine Serum	Sigma	Cat#F4135-500
ACK lysis buffer	Gibco	Cat#A10492-01
Sf-900 III Media	Invitrogen	Cat#12658019
ESF 921 Insect Cell Culture Medium	Expression Systems	Cat#96-001-01
16% paraformaldehyde	Fisher Scientific	Cat#Cat#50-980-487
Gentamicin	Gibco	Cat#15750078
Cell strainer - 70μM	Corning	Cat#431751
Propidium iodide	Thermo Fisher	Cat#P3566
Bovine Serum Albumin, Fraction V	Fisher	Cat#BP1605-100
Tween 20	Sigma	Cat#P9416
Influenza Vaccine Live, Intranasal FluMist® Quadrivalent 2021-2022	AstraZeneca	Lot#NK2075
Fluarix Quadrivalent 2021/2022 Formula	GlaxoSmithKline Biologicals	Lot#7LX9G
Ku, p70/p80	Fisher Scientific	Cat#50-253-496
LA/SS-B Human Recombinant	Protein Specialists	Cat#PRO-327
Recombinant Human IL-21	Peprotech	Cat#200-21
BS3	Thermo Fisher	Cat#A39266
Octyl Maltoside, Fluorinated, Anagrade	Anatrace	Cat#O310F
Insulin, Human Recombinant	Millipore Sigma	Cat#91077C
Small Nuclear Ribonucleoprotein 70kDa Human Recombinant	Novateni Bio	Cat#PT_72255
Kifunensine	Toronto Research Chemicals	Cat#K450000
RPMI 1640 Medium	Sigma	Cat#R8758-24X500ML
MEM non-essential amino acids	Gibco	Cat#11140050
Sodium pyruvate	Gibco	Cat#11360-070
1M HEPES	Gibco	Cat#15630-080
Penicillin-streptomycin	Gibco	Cat#15-140-163
Fetal Bovine Serum	Sigma	Cat#F4135-500
Human IL-21 (WT)	This study	N/A
Human IL-21 (Q116T)	This study	N/A
Human IL-21 (Q116I)	This study	N/A
Human IL-21 (Q116D)	This study	N/A
Human IL-21 (Q116A)	This study	N/A
Human IL-21 (Q116G)	This study	N/A
Human IL-21 (Q116S)	This study	N/A
Human IL-21 (Q116V)	This study	N/A
Human IL-21 (Q116L)	This study	N/A
MSA-humanIL-21 (WT)	This study	N/A
MSA-humanIL-21 (Q116T)	This study	N/A
MSA-humanIL-21 (Q116TI)	This study	N/A
Human IL-21 (N68Q)	This study	N/A
Critical commercial assays		
ExpiFectamine 293 Transfection Kit	Thermo Fisher	Cat#A14525
ELISA MAX™ Deluxe Set Human IFN-γ	BioLegend	Cat#430104
Nunc MaxiSorp ELISA plates, uncoated	BioLegend	Cat#423501
Chromogenic Endotoxin Quant Kit	Pierce	Cat#A39552
High Capacity NoEndo Columns	Protein Ark	Cat#Gen-NoE48HC
Human Granzyme B DuoSet ELISA	R&D	Cat#DY2906-05
ELISA MAX™ Deluxe Set Human IL-17A	BioLegend	Cat#433914
CellTrace™ Violet Cell Proliferation Kit	Thermo Fisher	Cat#C34557
1-Step™ Ultra TMB-ELISA Substrate Solution	Thermo Fisher	Cat #34028

(*Continued on next page*)

Continued		
REAGENT or RESOURCE	SOURCE	IDENTIFIER
96 Well V-Bottom 2mL Polypropylene Deep Well Plate	Corning	Cat#3960
MilliporeSigma™ Millicell™ Culture Plate Inserts	Fisher Scientific	Cat#PICM01250
B Cell Isolation Kit II, human	Miltenyi Biotech	Cat#130-091-151
Quantifoil R1.2/1.3 Micromachined Holey Carbon Grids	SPI	Cat#4220G-XA
C-Clip (100x)	Thermo Fisher	Cat#1036171
C-Clip Ring (100x)	Thermo Fisher	Cat#1036173
Deposited data		
IL-21 Receptor Complex Crystal Structure	PDB	8ENT
IL-21 Receptor Complex Electron Microscopy	EMDB	EMD-28278
Experimental models: cell lines		
Human: Expi293F	Thermo Fisher	Cat#A14528
Human: YT-1	RRID	Cat#CVCL_EJ05
Insect: Spodotera frugiperda (Sf9)	ATCC	Cat#CRL-1711
Insect: Trichoplusia ni (Hi5)	Expression Systems	Cat#94-002F
Recombinant DNA		
pD649	ATUM	Cat#PD649
pAcGP67a	BD	Cat#554756
pD649-hIL21-6xHis	This study	N/A
pD649-hIL21(N68Q)-6xHis	This study	N/A
pD649-hIL21(Q116T)-6xHis	This study	N/A
pD649-hIL21(Q116I)-6xHis	This study	N/A
pD649-hIL21(Q116D)-6xHis	This study	N/A
pD649-hIL21(Q116A)-6xHis	This study	N/A
pD649-hIL21(Q116G)-6xHis	This study	N/A
pD649-hIL21(Q116S)-6xHis	This study	N/A
pD649-hIL21(Q116L)-6xHis	This study	N/A
pD649-hIL21(Q116V)-6xHis	This study	N/A
pD649-MSA-hIL21-6xHis	This study	N/A
pD649-MSA-hIL21(Q116T)-6xHis	This study	N/A
pD649-MSA-hIL21(Q116I)-6xHis	This study	N/A
pAcGP67a-IL21R(N78Q/N85Q/N106D/N116Q)-6xHis	This study	N/A
pAcGP67a-gammaDel32-6xHis	This study	N/A
pD649-MUF-6xHis	This study	N/A
Software and algorithms		
FlowJo v10.5	Tree Star	RRID: SCR_008520
GraphPad Prism v9.3.0	GraphPad Software	RRID: SCR_002798
Phenix	Liebschner et al.[43]	RRID: SCR_014224
Coot	Emsley et al.[44]	RRID: SCR_014222
UCSF ChimeraX	Goddard et al.[45]	RRID: SCR_015872
PISA	Krissinel and Henrick[46]	RRID: SCR_015749
SBGrid	Morin et al.[47]	RRID: SCR_003511
SerialEM v4.0.0	Mastronarde et al.[48]	RRID: SCR_017293
CryoSPARC v3.3.2	Punjani et al.[49]	RRID: SCR_016501

CellPress
OPEN ACCESS

RESOURCE AVAILABILITY

Lead contact

Further information and requests for resources and reagents should be directed to and will be fulfilled by the lead contact, K. Christopher Garcia (kcgarcia@stanford.edu).

Materials availability

All unique reagents generated in this study are available from the lead contact with a completed Materials Transfer Agreement.

Data and code availability

- Structures and coordinates have been deposited in the Protein DataBank with identification number PDB: 8ENT. X-ray diffraction images have been deposited in the SBGrid DataBank with accession 974. Cryo-EM maps have been deposited to the Electron Microscopy DataBank (EMDB) under accession ID EMDB: EMD-28278.
- This paper does not report original code.
- Any additional information required to reanalyze the data reported in this work is available from the lead contact upon request.

EXPERIMENTAL MODEL AND SUBJECT DETAILS

Mammalian cell lines and culture conditions

For structural studies and signaling assays, IL-21 variants were produced in Expi293F cells (Gibco) cultured in Expi293 expression media (Gibco) at 37°C with 5% CO_2.

Signaling assays were conducted in YT-1 cells cultured in complete RPMI medium: RPMI 1640-glutaMAX (Gibco) containing 10% FBS (Fisher Scientific), 25mM HEPES (Gibco), 2mM pyruvate (Gibco), 4mM GlutaMAX (Gibco), non-essential amino acids (Gibco), and penicillin-streptomycin (Gibco) at 37°C with 5% CO_2.

Insect cell lines and culture conditions

For structural studies, baculovirus for IL-21R and γc was produced in *Spodoptera frugiperda* (Sf9) cells (ATCC) maintained in Sf-900 III medium (Gibco) with 10% FBS and GlutaMAX (Gibco).

Human primary cells and culture conditions

Peripheral mononuclear cells (PBMCs) from healthy donors were obtained from Stanford Blood Bank. They were cultured in complete RPMI at 37°C with 5% CO_2.

Human tonsil organoid culture

Tonsil tissue was collected from consented individuals undergoing surgery for hypertrophy, recurrent tonsillitis, or obstructive sleep apnea in accordance with the Stanford University Institutional Review Board.[28] Tonsil tissue was dissected and disrupted into a suspension through a 100μM strainer prior to cryopreservation. Cryopreserved tonsil samples were thawed and prepared as tonsil organoids by resuspension at 6×10^7 cells per ml, with 100μL cell mixtures cultured in permeable culture plate inserts (MilliporeSigma) in a 12-well plate.[28]

METHOD DETAILS

Protein production and purification

For crystallographic studies, a glycan-reduced mutant of human IL-21 (N68Q) was cloned into the pD649 mammalian expression vector containing a C-terminal 6xHis-tag. DNA was transiently transfected into Expi-293F cells (Thermo Fisher) using Expifectamine transfection reagent (Thermo Fisher). Supernatant was harvested 4 days post transfection. A glycan-reduced mutant of the human IL-21R extracellular domain (N78Q/N85Q/N106D/N116Q) was cloned into the pAcGP67a baculoviral vector with a C-terminal 6xHis-tag. Baculovirus was prepared by co-transfection of BestBac™ DNA (Expression Systems) and pAcGP67a DNA into *Spodoptera frugiperda* (Sf9) cells. P0 virus was harvested from the supernatant 6 days post-transfection. SF9 cells were infected with P0 viral supernatant at a 1:1000 dilution and P1 virus was harvested from the supernatant 6 days post infection. P2 virus was generated by the same procedure. Baculovirus was used to infect *Trichoplusia ni* (Hi5) cells. Supernatant was harvested from Hi5 cells 72 h after infection. Supernatant containing IL-21 and IL-21R were co-incubated with Ni-NTA resin (QIAGEN) for 4 h prior to elution with 0.2M imidazole followed by purification by size-exclusion chromatography (SEC) on a Superdex 200 column (GE). Finally, human common-gamma extracellular domain with an N-terminal truncation of 32 residues was cloned into pAcGP67a for baculovirus generation and Hi5 cells were infected, with the addition of 5μM kifunensine to the culture media. Supernatant was harvested from Hi5s 72 h after infection and purified by SEC on a Superdex 200 column.

For signaling studies, engineered variants of human IL-21 were cloned into the pD649 vector containing a C-terminal 6xHis-tag. DNA was transfected into Expi-293F cells as described above, and protein was purified from supernatant as described above.

Crystallization, data collection, and refinement

Following purification, human γc was deglycosylated by overnight incubation with Endo Hf (New England Biolabs) and purified by SEC on a Superdex 200 column. Deglycoslyated γc was mixed at an equimolar ratio to the copurified IL-21/IL-21R complex. The ternary complex was concentrated to 14 mg/mL and treated with carboxypeptidase A and B. Crystals of the IL-21 ternary complex were grown by sitting drop vapor diffusion with 100 nL of the complex, mixed with an equal volume of 0.2M magnesium chloride hexahydrate, 0.1M Tris pH 8, 20% PEG 6000, and crystals were harvested and cryoprotected using 30% ethylene glycol.

Diffraction data was collected at Stanford Synchrotron Radiation Laboratory beamline 12-2. Data were indexed, integrated, and scaled to 2.8 Å resolution using XDS.[50] The structure was solved by molecular replacement in Phaser[51] using models derived from structures of the IL-21/IL-21R binary complex (PDB: 3TGX)[17] and γc from the IL-2 quaternary complex (PDB: 2B5I),[20] identifying four copies of IL-21, four copies of IL-21R, and three copies of γc in the asymmetric unit. The structure was completed by iterative cycles of rebuilding and refinement in Coot[44] and Phenix.[43] Noncrystallographic symmetry restraints were used in refinement. Data collection and refinement statistics are presented in Table S1. The crystal structure has been deposited in the RCSB protein databank with accession code 8ENT. All crystallographic software was installed and configured by SBGrid.[47]

Cryo-electron microscopy

The same protein purification procedure used for crystallography was used to prepare IL-21 receptor complex for cryo-EM. Inspection of the crystal structure determined that lysines within crosslinking distance for BS^3 (11.4 Å) are present at the IL-21–IL-21R, IL-21–γc, and IL-21R–γc, but not the IL-21R—IL-21R, interfaces. The receptor complex was concentrated to 1 mg/mL and cross-linked with 2mM BS^3 (Thermo Fisher) as per manufacturer instructions. The cross-linked complex was purified by SEC on a Superdex 200 column. The indicated fractions (Figure S2B) were pooled and concentrated to 3 mg/mL. Samples were mixed with 0.01% fluorinated octyl maltoside (Anatrace) and applied to a glow-discharged Quantifoil (1.2/1.3) grid. The grids were blotted for 2 s at 100% humidity and plunge-frozen in liquid ethane using a GP Plunge Freezer (Leica). The grids were imaged on a 200 keV GlaciosTM cryo-transmission electron microscope (Thermo Fisher) equipped with a K3 camera (Gatan) at Stanford University. Data were collected at a nominal magnification of 45,000× in super-resolution counting mode, corresponding to a physical pixel size of 1.15 Å. Each movie was collected for a total of 5 s with 0.1 s exposure per frame at an exposure rate of ~13 electrons/pixel/s and a defocus range between −1.0 and −2.5 μm, using SerialEM[48] with beam image shift to collect 9 images from 9 holes per stage shift and focus.

Cryo-EM image processing and data processing

2D classifications and 3D reconstructions were performed using cryoSPARC v3.3.2. Reference-free 2D classification was performed on 1,444,277 particles. 2D classes were manually sorted into 2:2:2, 2:2, or 1:1:1 classes for further rounds of 2D sorting. 2D classes for each group were used to generate *ab initio* models. The 2:2:2 *ab initio* model was used in iterative rounds of 3D sorting against the full particle set. The 2:2:2 volume was further refined using non-uniform and local refinement, which resulted in a class with 57,295 particles and a resolution of 3.7 Å. The 2:2:2 crystal structure was docked into the 2:2:2 volume using ChimeraX. The resulting model had a map-model FSC of 4.2 Å at a 0.5 cutoff. Cryo-EM statistics are presented in Table S3.

Signaling assays in YT-1 cells

For phospho-flow cytometry experiments in human cell lines, YT-1 cells were plated in a 96-well plate (100,000 cells/well) and stimulated with wild-type or engineered IL-21 for 20 min at 37°C. Cells were fixed with 1.5% paraformaldehyde for 10 min at room temperature, followed by permeabilization with 100% methanol for 30 min at −20°C. Cells were stained with Alex Fluor 647 or Alexa Fluor 488 conjugated anti-STAT3 (pY705) antibody (BD) for 1 h at room temperature in FACS buffer (PBS pH 7.2, 1% FBS and 2mM EDTA). Fluorescence intensities were measured using a CytoFlex flow cytometer (Beckman Coulter), and dose-response curves were plotted and fit in Prism 9.3.0 (GraphPad).

Signaling assays in human PBMCs

Human peripheral blood mononuclear cells (PBMCs) were isolated from donor samples (Stanford Blood Center) and cryopreserved until use. For activation of human T cells, 6-well plates were pre-coated with 2 μg/mL anti-human CD3 clone OKT3 (BioLegend) in PBS and incubated overnight at 4°C. Frozen PBMCs from human donors were recovered and resuspended in complete RPMI with 5 μg/mL anti-CD28 (BioLegend) and added to the coated plate and incubated for 48 h at 37°C. After 48 h, cells were harvested from the plate, washed with complete RPMI and rested without stimulation overnight at a density of 1-2E6 cells/mL at 37°C in complete RPMI. To identify CD4 and CD8 T cells, Fc receptors were blocked with Human TruStain FcX (BioLegend), and cells were stained with anti-human CD8 Brilliant Violet 605 (BioLegend) and anti-human CD4 Pacific Blue (BioLegend) for 15 min at 4°C. Cells were washed with FACS buffer and plated in a 96-well plate (200,000 cells/well) and stimulated with wild-type or engineered IL-21 for 20 min at 37°C. Cells were fixed with paraformaldehyde and permeabilized with methanol, stained for pSTAT3 and pSTAT1, and assayed by flow cytometry as described above.

Tonsil organoid culture

Tonsil organoids were vaccinated with FluMist live attenuated influenza vaccine (LAIV) from the 2021–2022 flu season and stimulated with 10nM IL-21, IL-21 variant, or IL-21R blocking scFV MUF.[28] On day 4, 500 μL media with cytokine was supplemented. On day 7, cells from the organoid were harvested and counted. 1 million cells per condition were used for cell surface phenotyping by flow cytometry. Cells for phenotype analysis were stained for 30 min at 4°C with a panel containing TruStain FcX (BioLegend), anti-human CXCR5 FITC, anti-human CD27 PE/Cyanine 7, anti-human CD19 PerCP/Cyanine5.5, anti-human CD38 APC, anti-human CD4 Pacific Blue, anti-human CD45 Blue Violet 605, anti-human PD-1 Blue Violet 785 (all antibodies BioLegend) in FACS buffer. Cells were washed and resuspended in FACS buffer containing propidium iodide (Thermo Fisher) to stain dead cells. Cell subsets were analyzed by flow cytometry on a CytoFlex (Figures S5A and S5B). Flow cytometry data was analyzed by FlowJo v10.5 (Tree Star) and cell counts were plotted using Prism v. 9.3.0 (GraphPad).

Antibody detection by ELISA

Supernatants from the tonsil cultures were harvested for analysis by ELISA. Nunc MaxiSorp ELISA plates (BioLegend) were coated with 1 μg/mL inactivated influenza vaccine from the 2021–2022 season or 1 μg/mL HA variants. For autoantibody ELISAs, plates were coated with 1 μg/mL human dsDNA (Sigma), Ku, p70/p80 (Fisher Scientific) or LA/SS-B (Protein Specialists). After overnight incubation at 4°C, plates were blocked with 1% bovine serum albumin (Fisher). Samples were prepared at specified dilutions in PBS before being added to the plate and incubated for 1 h at room temperature, followed by incubation with anti-human Fc-IgG-HRP secondary antibody. The plates were incubated with secondary antibody for 1 h at room temperature. TMB substrate was mixed per manufacturer instructions (Thermo Fisher) to develop the plate, and the reaction was stopped after 10 min with 2M H_2SO_4 and analyzed on a SpectraMax i3X Multi-Mode Microplate Reader (Molecular Devices).

The broadly neutralizing antibody MEDI8852 was used to generate standard curves to interpolate the quantity of tonsil antibodies binding to influenza[52] (Figure S5D). Samples were analyzed at 1:10 or 1:100 dilutions and interpolated using a standard curve (Figures S5D and S5E), or serially diluted to generate binding curves (Figures S5C and S5G) to calculate reciprocal IC50s and area under the curve (AUC). ELISAs were conducted as described above. Standard curves, interpolations, IC50s, and AUCs were calculated in Prism v. 9.3.0 (GraphPad).

Human B cell activation assays

For human B cell activation assays, human B cells were isolated from PBMCs using a B Cell Isolation Kit (Miltenyi Biotech). For short term activation assays, human B cells were cultured for 3 h with RPMI with or without 1 μg/mL anti-human CD40 (BioLegend), 100 ng/mL biotinylated anti-human lambda chain and anti-human kappa chain (Southern Biotech), and 100nM WT human IL-21, Q116T, or Q116I variant. 10 μg/mL neutravidin was added in the last minute of activation. For three-day activation assays, splenocytes were cultured for 3 days in RPMI with or without 100 ng/mL or 1 μg/mL anti-CD40, and 100 ng/mL biotinylated anti-lambda and anti-kappa cross-linked with neutravidin. Cells were washed and rested for 1 h without the activating antibody cocktail, then stimulated with 100 nM WT human IL-21, Q116T, or Q116I for 20 min at 37°C. For all activation assays, cells were fixed, permeabilized, and analyzed for phospho-S6, phospho-STAT3, and phospho-STAT1 activation by flow cytometry.

QUANTIFICATION AND STATISTICAL ANALYSIS

Flow cytometry data was analyzed using FlowJo v. 10.5 (Tree Star). Statistical analyses and dose-response curves were generated using Prism v. 9.3.0 (GraphPad). For dose-response analyses, a sigmoidal 4PL analysis was used in Prism. For analysis of fold-change IgG across human donors in tonsil organoid experiments, paired nonparametric ANOVA was used (Friedman's test). For analysis of fold-change cell counts across human donors in tonsil organoid experiments, Friedman's test was used.

Cell Reports, Volume *42*

Supplemental information

A structural blueprint for interleukin-21 signal modulation

Gita C. Abhiraman, Theodora U.J. Bruun, Nathanael A. Caveney, Leon L. Su, Robert A. Saxton, Qian Yin, Shaogeng Tang, Mark M. Davis, Kevin M. Jude, and K. Christopher Garcia

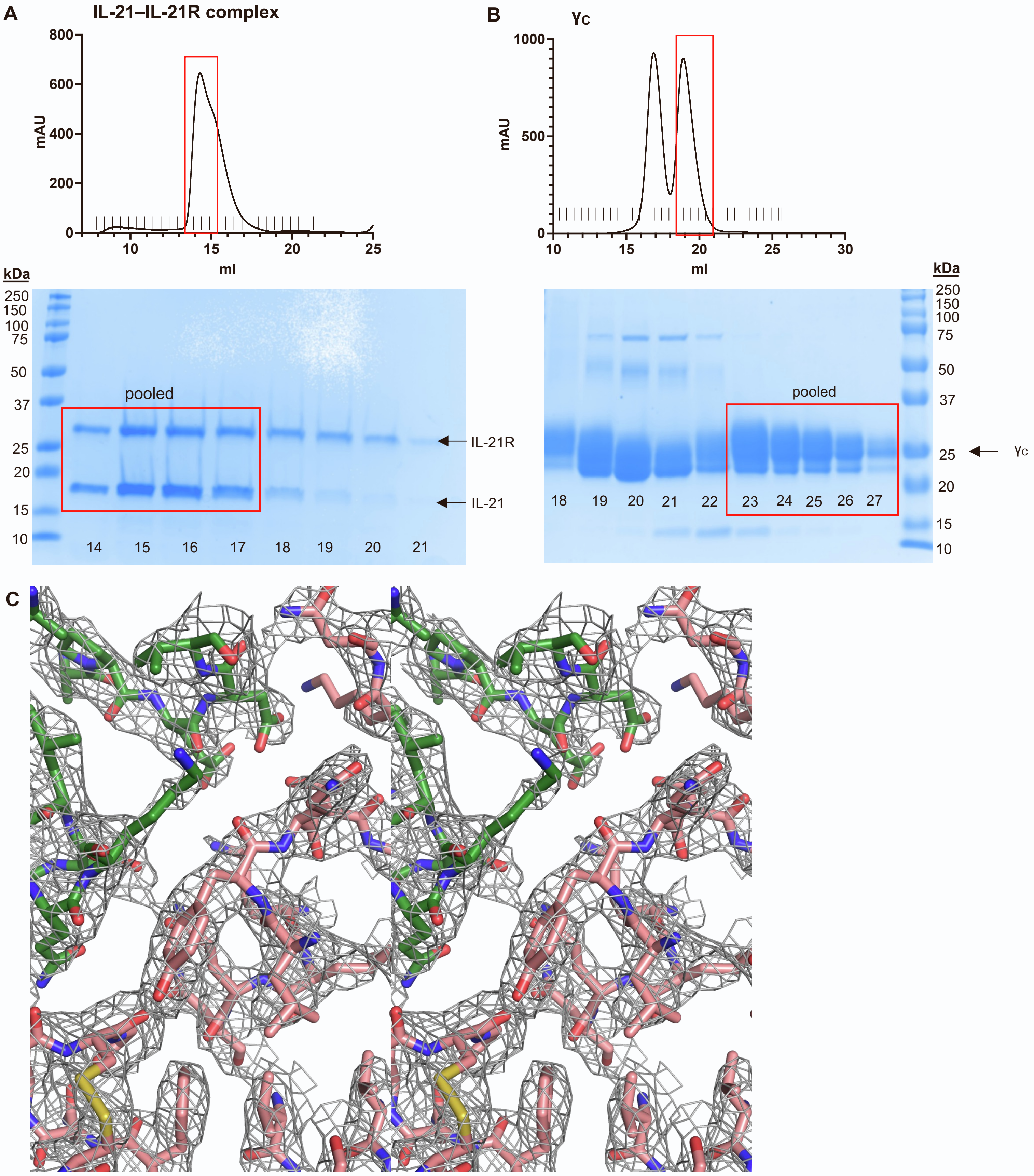

Figure S1. Structure of the IL-21 receptor complex, related to Figure 1

(A–B) Size exclusion chromatography UV traces for purified IL-21R and IL-21 (A) complex and γ_C (B) and accompanying Coomassie-stained SDS-PAGE gel. Fractions collected for crystallization trials are indicated in red. **(C)** Wall-eyed stereo view of site IIa interface. The final 2mFo-DFc map (shown in gray) is contoured at 1.0 σ. IL-21 shown in green, γc in pink.

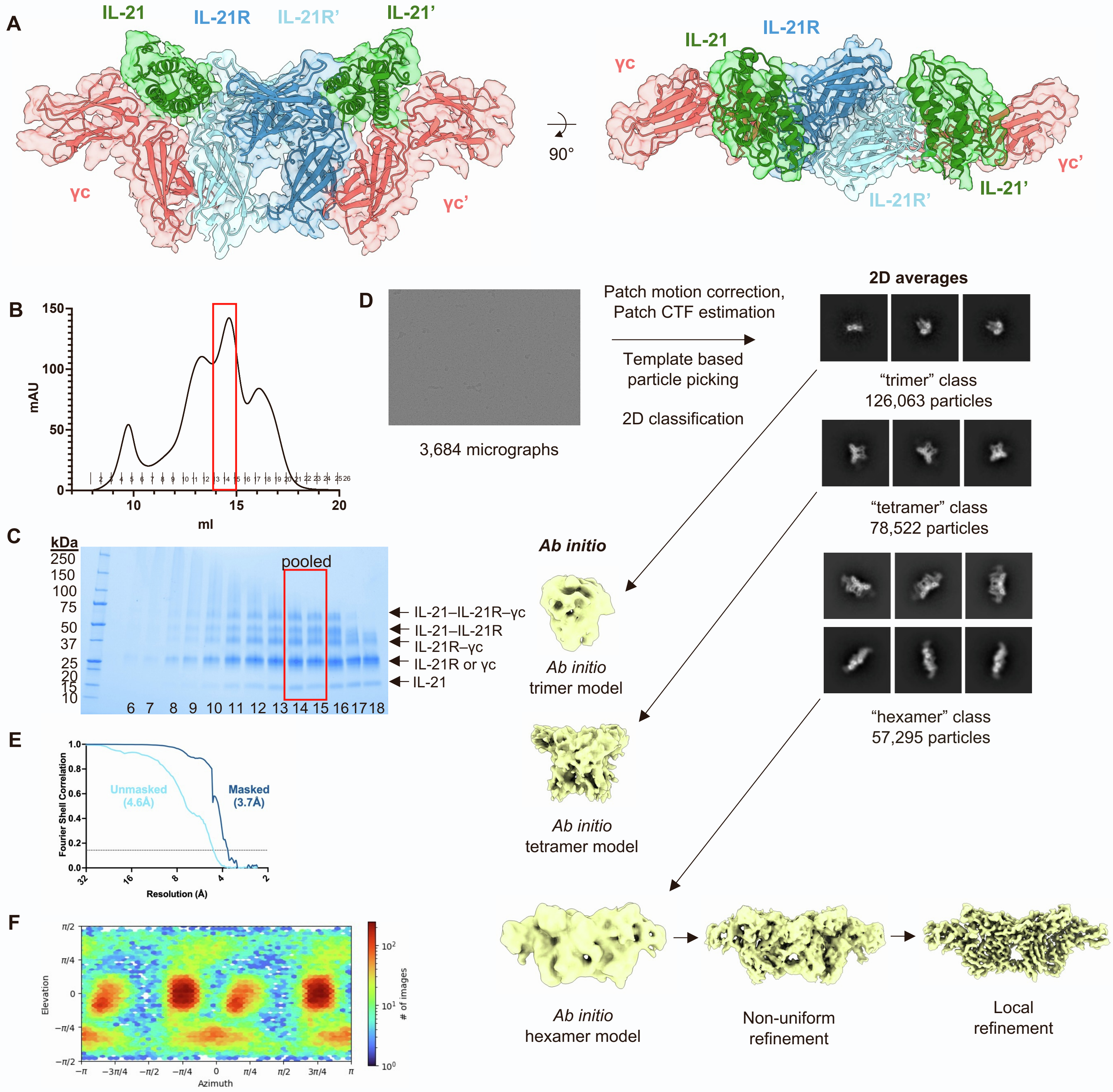

Figure S2. Cryo-electron microscopy of the IL-21 complex, related to Figure 1

(A) Final model of 2:2:2 IL-21:IL-21R:γ_C complex determined by cryoEM (shown in transparency, EMDB ID: EMD-28278) with crystallography model docked (shown as ribbon, PBD ID: 8ENT).

(B–C) Size exclusion chromatography UV trace for IL-21–IL-21R–γc complex after BS^3 cross-linking and accompanying Coomassie-stained SDS-PAGE gel. Fractions collected for cryo-EM studies are outlined in red.

(D) Cryo-EM data processing of the IL-21 complex, including representative micrograph from data acquisition. 2D class averages for hexamer, tetramer, and trimer classes and *ab initio* models generated for each class, followed by refinement scheme for the hexameric model.

(E) Fourier shell correlation (FSC) curve of final model.

(F) Orientational distribution of the complex reconstruction.

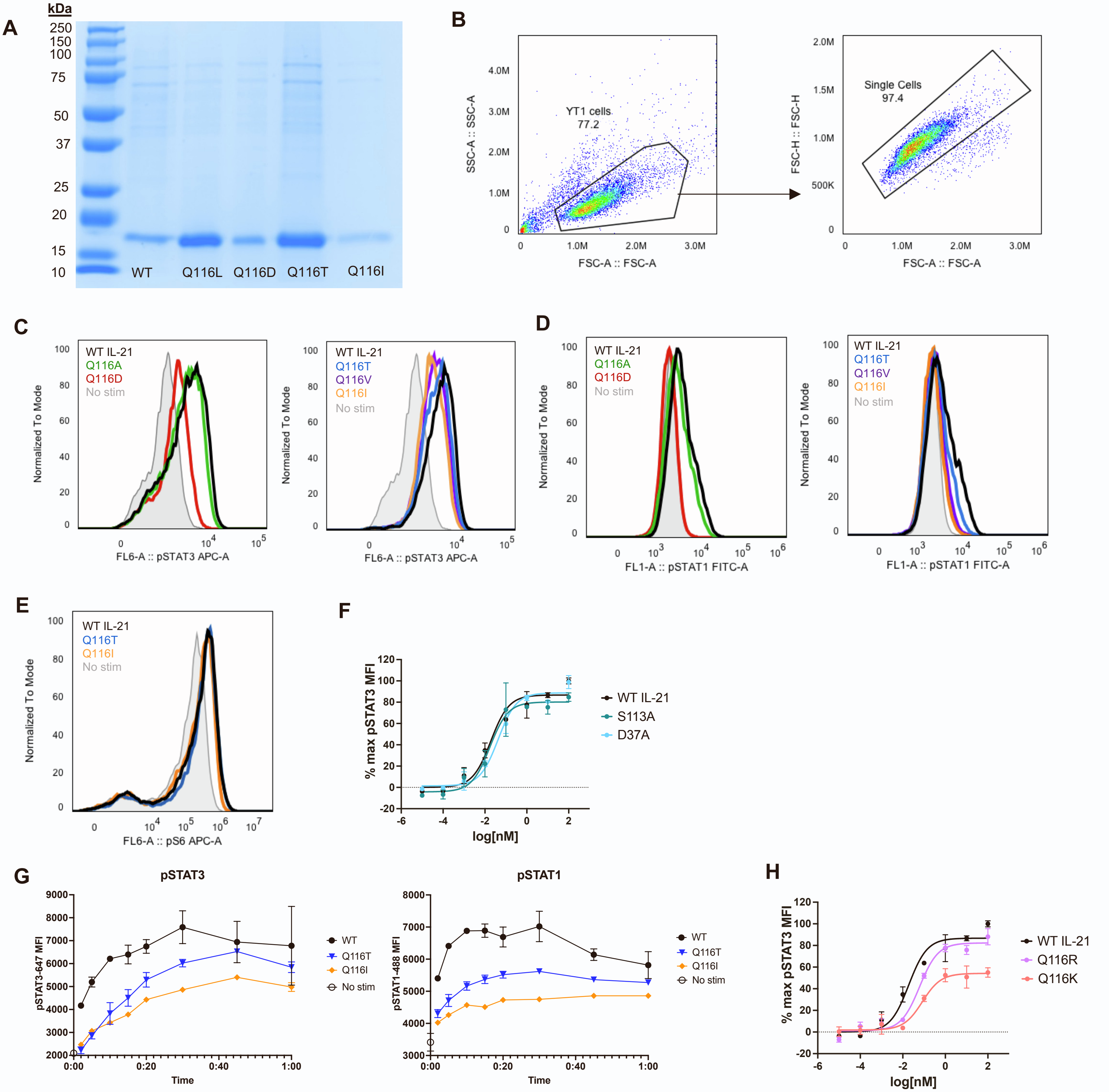

Figure S3. Design of human IL-21 partial agonists, related to Figure 3

(A) Coomassie-stained SDS-PAGE gel of His-tagged IL-21 variants purified by Ni-NTA.
(B) Representative gating on single YT-1 cells.
(C–D) Representative histograms of pSTAT3 (C) or pSTAT1 (D) intensity in YT-1 cell stimulated with wild-type IL-21 or variant.
(E) Representative histograms of pS6 intensity in YT-1 cells stimulated with wild-type IL-21 or variant.
(F) Dose-response curves for phopsho-STAT3 in YT-1 cells stimulated with wild-type IL-21 or the indicated variants for 20 minutes and analyzed by flow cytometry. Data are mean +/- SD for two replicates, shown as percent of maximal wild-type IL-21 MFI.
(G) Time-course signaling assays for intracellular phospho-STAT3 and phospho-STAT1 in YT-1 cells stimulated with 200nM IL-21 or variant for up to one hour. Data are mean +/- SD for two replicates.
(H) Dose-response curves for phopsho-STAT3 in YT-1 cells stimulated with wild-type IL-21 or the indicated variants for 20 minutes and analyzed by flow cytometry. Data are mean +/- SD for two replicates, shown as percent of maximal wild-type IL-21 MFI.

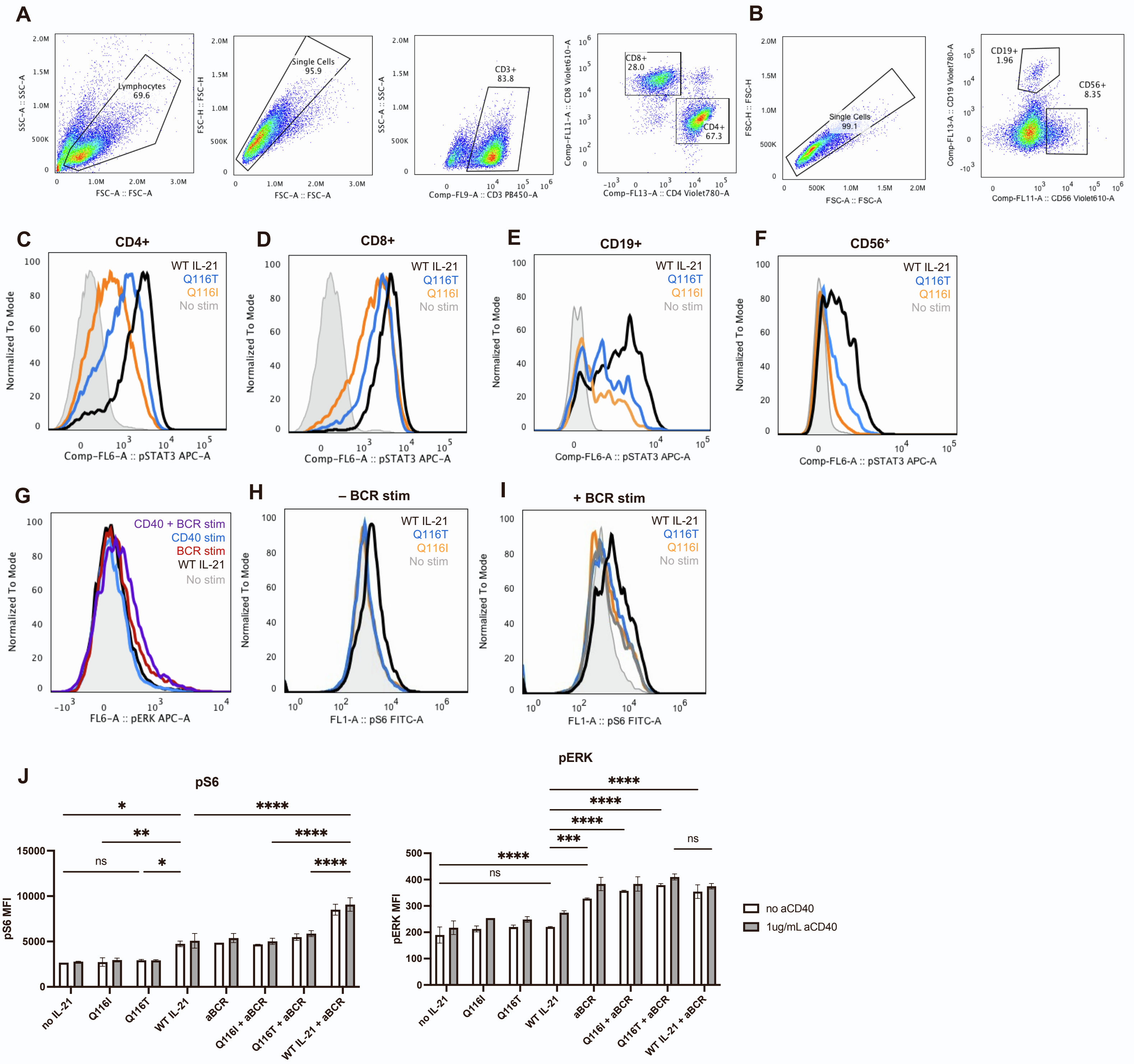

Figure S4. IL-21 signaling in primary human cells

(A–B) Representative gating of primary CD4⁺ and CD8⁺ T cells (A) or CD19⁺ and CD56⁺ cells from bulk human peripheral mononuclear cells (B).

(C–F) Representative histograms of pSTAT3 intensity in CD4⁺ (C), CD8⁺ (D), CD19⁺ (E) or CD56⁺ (F) cells stimulated with wild-type IL-21, Q116T, Q116I, or no cytokine.

(G) Representative histograms of pERK intensity of MACS isolated CD19⁺ cells stimulated with wild-type IL-21, BCR stimulation, or CD40 stimulation, corresponding to Figure 4B.

(H–I) Representative histograms of pS6 intensity of CD19⁺ cells stimulated with wild-type IL-21, Q116T, Q116I, or no cytokine with (H) or without (I) BCR stimulation, corresponding to Figure 4C.

(J) Bar graphs quantifying mean intensity of pS6 and pERK in B cells detected by flow cyometry, corresponding to heat maps in Figure 4B–C (N=2 biological replicates, * indicates $P \leq 0.05$, ** indicates $P \leq 0.01$, *** indicates $P \leq 0.001$, **** indicates $P \leq 0.0001$ by two-way ANOVA).

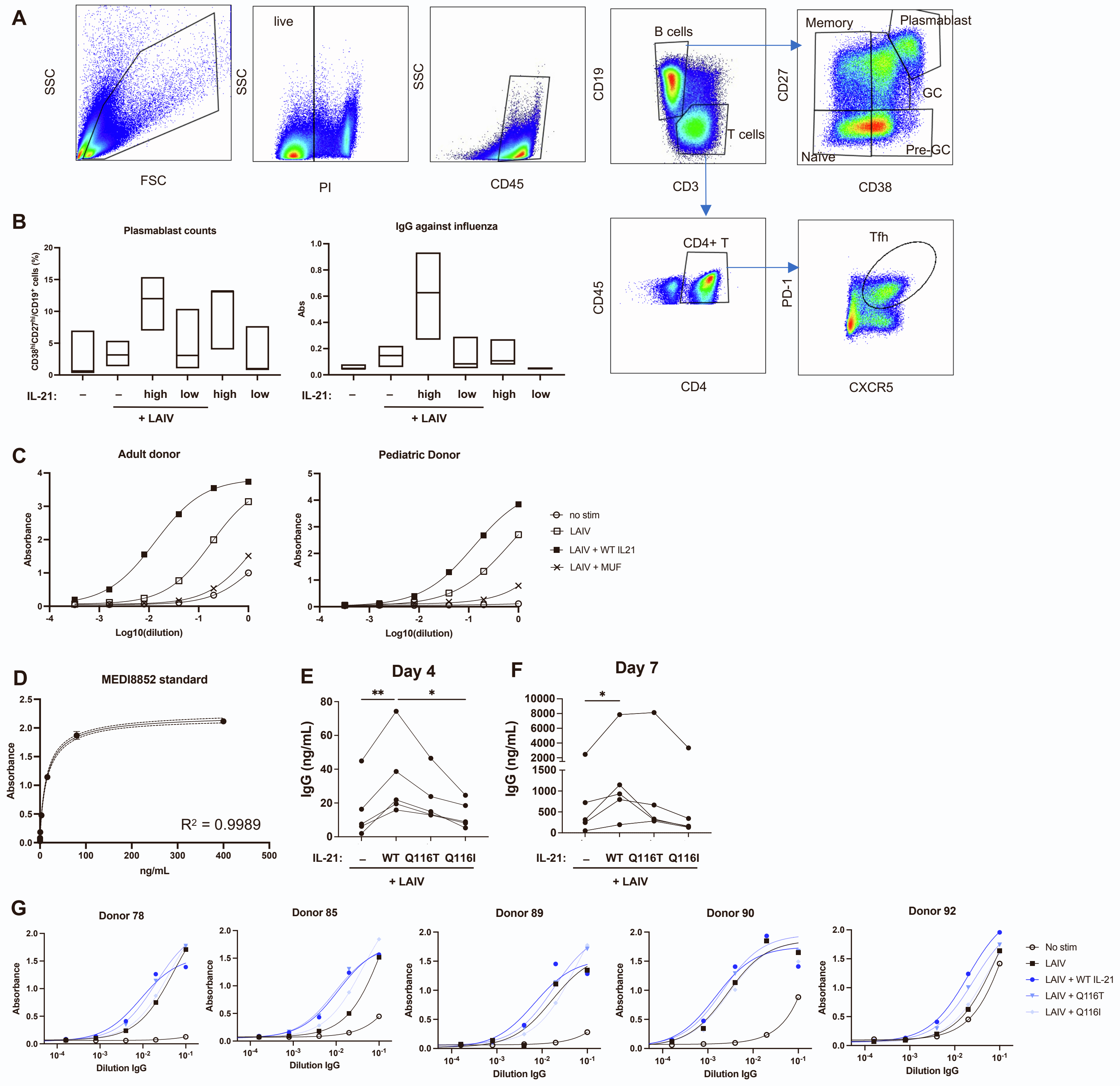

Figure S5. Characterization of IL-21 response in human tonsil organoids, related to Figure 4

(A) Representative gating of naïve B cells, memory B cells, germinal center B cells (GC), pre-germinal center B cells (pre-GC), plasmablasts, and T follicular helper (Tfh) cells in human tonsil organoids.

(B) Plasmablast frequency (% of total B cells) and flu-specific IgG detected by ELISA from tonsil organoids cultured for 7 days with or without live attenuated influenza virus (LAIV) and 50ng/ml (high dose) or 10ng/mL (low dose) IL-21 (N=3 human donors).

(C) Representative titrations of flu-specific IgG detected by ELISA in human tonsil organoids from an adult and pediatric donor.

(D) Representative standard curves derived from broadly neutralizing antibody MEDI8852 binding to whole flu used for quantification of flu-specific antibodies in human tonsil experiments.

(E–F) Flu-specific IgG quantified by ELISA from tonsil organoids vaccinated with LAIV and indicated IL-21 variant on day 4 (F) and day 7 (G). IgG was quantified using broadly neutralizing flu antibody. Raw IgG values plotted as ng/mL. * indicates P ≤ 0.05, ** indicates P ≤ 0.01 by ANOVA. (N=5 human donors.)

(G) Titrations of flu-specific IgG detected by ELISA in human tonsil organoids derived from 5 donors, stimulated with LAIV, wild-type IL-21, Q116T, or Q116I.

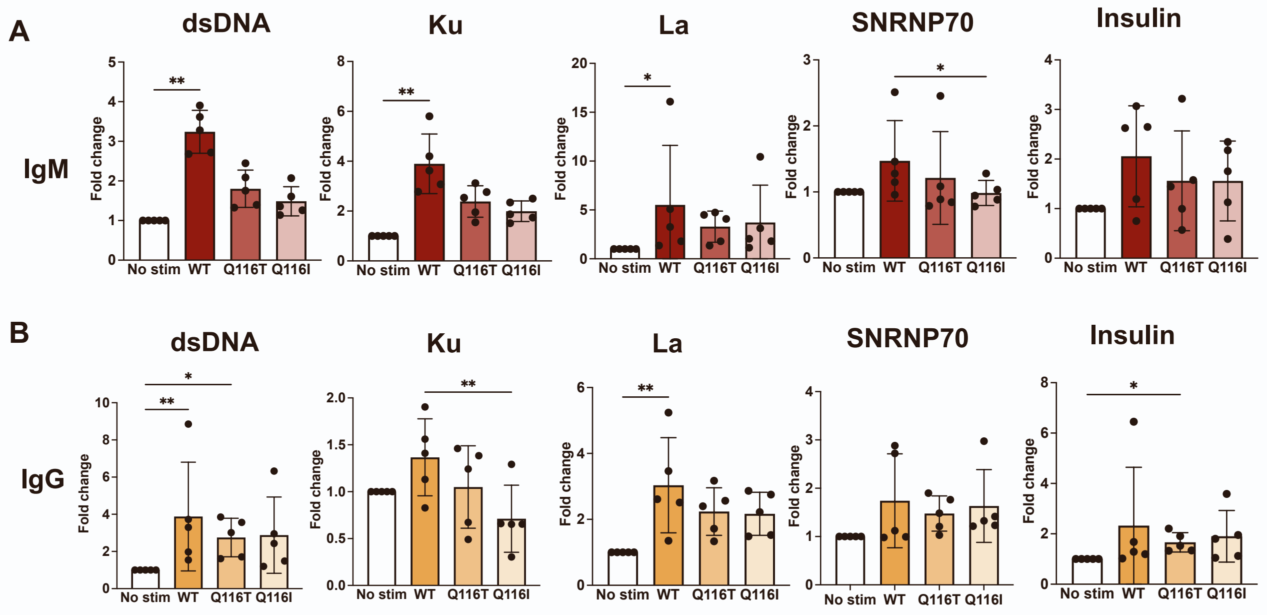

Figure S6. Autoantibody production is modulated by IL-21 variants

(A–B) IgM (A) and IgG (B) against self-antigens dsDNA, Ku and La, quantified by ELISA on day 7 in tonsils cultured with 100nM IL-21 or variant. Data are mean +/- SD for five human donors. * indicates P ≤ 0.05, ** indicates P ≤ 0.01 by one-way ANOVA.

Table S1. Data collection and refinement statistics, related to Figure 1

Protein	IL-21–IL-21R– γc complex
PDB ID	8ENT
**Data collection**	
Wavelength	0.9795
Resolution range (Å)	48.17 - 2.831 (2.932 - 2.831)
Space group	P1
a, b, c (Å)	66.167 66.464 162.684
α, β, ɣ	83.338 83.708 73.098
Total reflections	211574 (20618)
Unique reflections	57754 (5757)
Multiplicity	3.7 (3.6)
Completeness (%)	91.66 (88.39)
Mean I/sigma	4.22 (0.66)
R_{sym}	0.2143 (1.636)
R_{meas}	0.2506 (1.914)
R_{pim}	0.1292 (0.9883)
$CC_{1/2}$	0.985 (0.401)
CC*	0.996 (0.757)
**Refinement**	
Reflections used in refinement	57397 (5529)
Reflections used for R-free	1963 (194)
R_{work}	0.2784 (0.4586)
R_{free}	0.3120 (0.4967)
Number of non-hydrogen atoms	15852
macromolecules	15520
ligands	277
solvent	55
Protein residues	1888
RMS(bonds) (Å)	0.002
RMS(angles) (°)	0.6
Ramachandran favored (%)	94.72
Ramachandran allowed (%)	5.28
Ramachandran outliers (%)	0
Rotamer outliers (%)	3.76
Clashscore	13.37
Average B-factors:	74.21
macromolecules	74.11
ligands	84.98
solvent	48.56

Table S2. Key contacts in IL-21 receptor complex

Site IIa IL-21–IL-21R interface			
Hydrogen Bonds	γc	Dist. [Å]	IL-21
1	F:THR 105 [Oγ]	2.75	D:ASP 37 [Oδ]
2	F:GLN 127 [Nε]	3.12	D:SER 113 [Oγ]
3	F:GLN 127 [Oε]	3.52	D:GLN 116 [Nε]

Site IIb IL-21R–γc interface			
Hydrogen Bonds	γc	Dist. [Å]	IL-21R
1	F:ARG 183 [Nη]	3.17	E:GLU 136 [Oε]
2	F:SER 146 [Oγ]	2.81	E:GLU 170 [Oε]
3	F:GLN 147 [Nε]	2.77	E:LEU 156 [O]
4	F:SER 187 [Oγ]	3.13	E:SER 158 [Oγ]
5	F:SER 187 [Oγ]	2.78	E:LEU 156 [O]
6	F:GLN 147 [Oε]	3.12	E:LEU 156 [N]
7	F:SER 187 [Oγ]	3.72	E:SER 158 [N]
8	F:SER 190 [Oγ]	2.83	E:LEU 167 [N]
9	F:TYR 182 [Oη]	2.70	E:LYS 134 [Nζ]

Site III IL-21R–IL-21R interface			
Hydrogen Bonds	IL-21R	Dist. [Å]	IL-21R
1	H:HIS 24 [Nδ]	3.20	E:ASP 125 [Oδ]
2	H:HIS 53 [Nε]	2.43	E:ASP 122 [Oδ]
3	H:TRP 148 [N]	3.17	E:ARG 144 [O]
4	H:TRP 148 [N]	2.91	E:ASP 146 [O]
5	H:TRP 148 [Nε]	2.55	E:GLU 178 [Oε]
6	H:ALA 149 [N]	2.90	E:ARG 144 [O]
7	H:THR 61 [Oγ]	2.92	E:HIS 63 [Nε]
8	H:ASP 122 [Oδ]	2.70	E:HIS 53 [Nε]
9	H:ASP 125 [Oδ]	3.00	E:HIS 24 [Nδ]
10	H:ARG 144 [O]	3.10	E:TRP 148 [N]
11	H:ARG 144 [O]	2.67	E:ALA 149 [N]
12	H:ASP 146 [O]	2.73	E:TRP 148 [N]
13	H:GLU 178 [Oε]	2.77	E:TRP 148 [Nε]

Site III IL-21R–IL-21R interface			
Salt Bridges	IL-21R	Dist. [Å]	IL-21R
1	H:HIS 24 [Nδ]	3.20	E:ASP 125 [Oδ]
2	H:HIS 53 [Nε]	2.43	E:ASP 122 [Oδ]
3	H:ASP 122 [Oδ]	2.70	E:HIS 53 [Nε]
4	H:ASP 122 [Oδ]	2.95	E:HIS 53 [Nε]
5	H:ASP 125 [Oδ]	3.00	E:HIS 24 [Nδ]

Table S3. Cryo-EM data collection, refinement, and validation statistics

**Protein**	**IL-21–IL-21R– γc complex**
**EMD**	EMD-28278
**Data collection and processing**	
Magnification	45,000
Voltage (keV)	200
Electron exposure ($e^-/Å^2$)	50
Defocus range (μm)	-1.0 to -2.5
Pixel size (Å)	1.15
Symmetry imposed	C2
Initial particle images	1,444,277
Final particle images	57,295
Map resolution FSC threshold (Å)	0.143
Map resolution (Å)	3.7
**Model Fitting**	
Model used (PDB)	8ENT
Model resolution FSC threshold (Å)	0.5
Model resolution (Å)	4.2
